# Sesquiterpenoids from Chinese Agarwood Induced by Artificial Holing

**DOI:** 10.3390/molecules21030274

**Published:** 2016-02-26

**Authors:** Wei Li, Ge Liao, Wen-Hua Dong, Fan-Dong Kong, Pei Wang, Hao Wang, Wen-Li Mei, Hao-Fu Dai

**Affiliations:** 1 Key Laboratory of Biology and Genetic Resources of Tropical Crops, Ministry of Agriculture, Institute of Tropical Bioscience and Biotechnology, Chinese Academy of Tropical Agricultural Sciences, Haikou 571101, Hainan, China; liwei@itbb.org.cn (W.L.); lg8goelia@sina.cn (G.L.); dongwenhua@itbb.org.cn (W.-H.D.); kongfandong@itbb.org.cn (F.-D.K.); wangpei@itbb.org.cn (P.W.); Hao.Wang@uni-duesseldorf.de (H.W.); 2 Hainan Engineering Research Center of Agarwood, Haikou 571101, Hainan, China

**Keywords:** sesquiterpenoid, Chinese agarwood induced by artificial holing, *Aquilaria sinensis*, AChE inhibition activity, antibacterial activity

## Abstract

Two new sesquiterpenoids, 3-oxo-7-hydroxylholosericin A (**1**) and 1,5;8,12-diepoxy-guaia-12-one (**2**), together with seven known sesquiterpenoids **3**–**9**, were isolated from Chinese agarwood induced by artificial holing originating from *Aquilaria sinensis* (Lour.) Gilg. Their structures were identified by spectroscopic techniques (UV, IR, 1D and 2D NMR) and MS analyses. The absolute configuration of compound **1** was determined by comparison of its measured CD curve with that of calculated data for **1** and *ent*-**1**. The NMR data of **3** were reported in this study for the first time. Compounds **1**, **2**, **4**–**6**, together with the EtOAc extract showed moderate inhibitory activities against acetylcholinesterase, and compounds **4**–**6**, **8** exhibited antibacterial activities against *Staphylococcus aureus* or *Ralstonia solanacearum*.

## 1. Introduction

**Figure 1:**
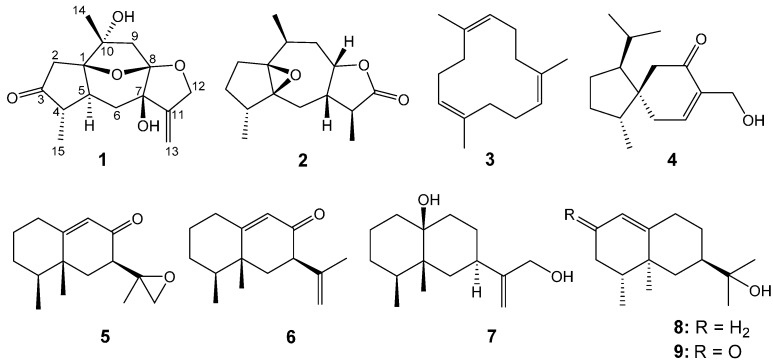
Chemical structures of compounds **1**–**9**.

Chinese agarwood is the resinous wood from the tree of *Aquilaria sinensis* (Lour.) Gilg (Thymelaeaceae), which is known as a famous traditional medicine, and has been reported as a folk medicine to possess various functions as sedative, analgesic, and digestive, *etc.* [1,2]. However, Chinese agarwood cannot be generated in healthy wood tissues of *A. sinensis* but may be produced when an *A. sinensis* tree is injured by lightning strikes, physical cutting, eaten by moths, burnt, bacterial infections or chemical stimulation, *etc.* [3,4]. The previous phytochemical research on wild Chinese agarwood from *A. sinensis* revealed the main constituents of Chinese agarwood were sesquiterpenoids and 2-(2-phenylethyl)chromones [4,5,6]. It is reported that some sesquiterpenoids and 2-(2-phenylethyl)chromones showed acetylcholinesterase inhibition activities, together with some antibacterial activities [5,7,8]. For better understanding of the chemical components of artificially induced Chinese agarwood, a phytochemical investigation was carried out on Chinese agarwood induced by artificial holing from *A. sinensis*, which led to the isolation of two new sesquiterpenoids **1**–**2** with unusual guaiane skeletons, together with seven known sesquiterpenoids **3**–**9** (Figure 1). The 1D and 2D NMR data of the known compound **3** are reported for the first time. The acetylcholinesterase inhibition activities, together with the antibacterial activities against *S. aureus* and *R. solanacearum* of these isolates were investigated *in vitro*.

## 2. Results and Discussion

**Figure 2:**
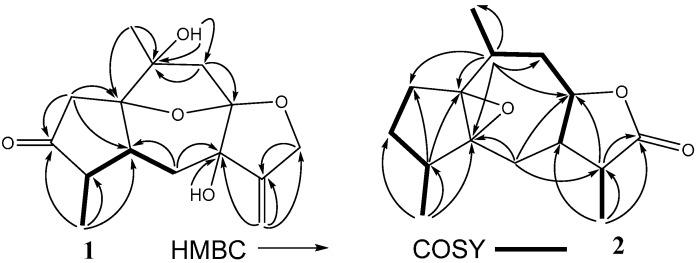
Key HMBC and ^1^H-^1^H COSY correlations of compound **1** and **2**.

**Figure 3:**
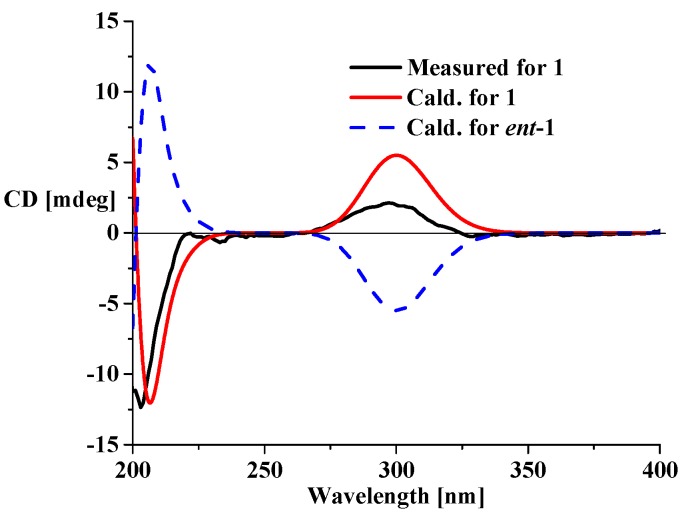
Measured CD curves of **1** and calculated CD curves of **1** and *ent*-**1**.

Compound **1** was obtained as a colorless oil, and its molecular formula was established as C_15_H_20_O_5_ by the HR-ESI-MS molecular peak at *m*/*z* [M + CF_3_COO]^−^ 393.1166 (calcd. 280.1311 for C_15_H_20_O_5_) (Supplementary Materials), with six degrees of unsaturation. The IR spectrum clearly demonstrated the presence of hydroxy (3434 cm^−1^), carbonyl (1630 cm^−1^) and olefinic bond (1597 cm^−1^) groups. The ^1^H-NMR spectroscopic data (Table 1) of **1** showed two methyls at δ_H_ 1.35 (3H, s, H-14) and 1.06 (3H, d, *J* = 7.3 Hz, H-15), one oxygenated methylene [δ_H_ 4.62 (1H, dt, *J* = 12.8, 2.4 Hz, H-12a) and 4.42 (1H, dt, *J* = 12.8, 2.4 Hz, H-12b)], and one terminal olefinic methylene [δ_H_ 5.28 (1H, br t, *J* = 2.4, H-13a) and 5.07 (1H, br t, *J* = 2.4 Hz, H-13b)]. The ^13^C-NMR spectrum (Table 1) of **1** exhibited 15 carbon signals, composed of two methyls, five methylenes, two methines and six quaternary carbons, indicative of a possible sesquiterpenoid skeleton. Detailed comparison of the NMR data with those of holosericin A [9] revealed that they shared the same guaiane skeleton with an ether bridge linking C-1 and C-8, and the two compounds differed only by a carbonyl (δ_C_ 219.0) and an oxygenated quarternary carbon (δ_C_ 75.1) in **1** replacing the corresponding aliphatic methylene and methine in holosericin A. This assignment was further proved by key HMBC correlations (Figure 2) from H_3_-15 (δ_H_ 1.06) to C-3 (δ_C_ 219.0), C-4 (δ_C_ 48.6) and C-5 (δ_C_ 44.7), and from H_2_-13 (δ_H_ 5.28, 5.07) to C-7 (δ_C_ 75.1), C-11 (δ_C_ 151.1) and C-12 (δ_C_ 71.3). Thus, the planar structure of compound **1** was established as 3-oxo-7-hydroxylholosericin A. Detailed analysis of ROESY and NOE difference spectra revealed that the relative configuration of the stereogenic centers C-1, C-4, C-5, C-8, and C-10 of compound **1** were the same as those of holosericin A; the additional hydroxyl located at C-7 was on the same side of the 7-membered ring system as the epoxy group based on the observation of the hydroxy proton (δ_H_ 5.16) enhancement when the H-4 (δ_H_ 2.16) were irradiated in the NOE difference experiment. The measured CD spectrum of **1** exhibited a characteristic cotton effect around 300 nm due to n→π* transition of the ketone group. In order to determine the absolute configuration of **1**, ECD calculations of **1** and *ent*-**1** using the time-dependent density functional theory (TD-DFT) method at the B3LYP/6-31G(d) level [10] was performed. The preliminary conformational distribution search was performed by HyperChem 7.5 software. The corresponding minimum geometries were further fully optimized by using DFT at the B3LYP/6-31G(d) level as implemented in the Gaussian 03 program package. The ECD calculations were performed after optimization of the selected conformers at the B3LYP/6-31G(d) levels [11]. The results showed that the measured CD curve matched well with the calculated ECD for **1** and was opposite to that of *ent*-**1** (Figure 3), indicating the 1*R*,4*S*,5*S*,7*R*,8*R*,10*R-*configuration.

**Figure 4:**
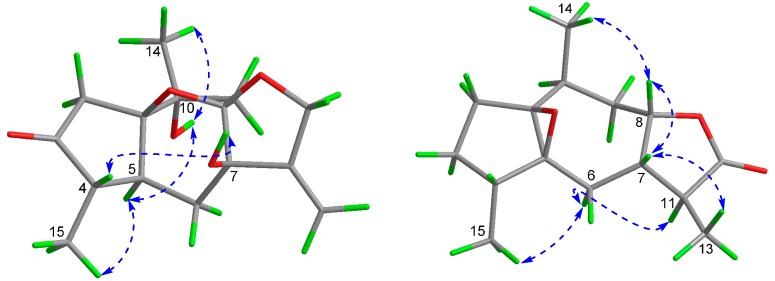
Key ROESY correlations of compound **1** and **2**.

Compound **2** was isolated as a colorless oil. The molecular formula was determined to be C_15_H_22_O_3_ on the basis of HR-ESI-MS (*m*/*z* 250.1563 [M]^+^, calcd. 250.1569 for C_15_H_22_O_3_) (Supplementary Materials), indicating five degrees of unsaturation. The ^13^C, DEPT NMR spectra along with the HSQC experiment indicated the presence of three methyls, four methylenes, five methines and three quaternary carbons, a carbonyl included. The structural units of C-2–C-3–C-4–C-15, C-11–C-13 and C-7–C-8–C-9–C-10–C-14 were undoubtedly determined by the ^1^H-^1^H COSY spectrum. The fragment of C-5–C-1(C-2)–C-10(C-14)–C-9–C-8 was determined by the HMBC correlations from H-10 (δ_H_ 2.59) to C-1(δ_C_ 73.8)/C-2(δ_C_ 29.6)/C-5(δ_C_ 72.4)/C-8(δ_C_ 80.7)/C-9(δ_C_ 35.2)/C-14(δ_C_ 17.0). The HMBC correlation from H-15 (δ_H_ 0.95) to C-3(δ_C_ 26.4)/C-4(δ_C_ 39.6)/C-5, from H-6 (δ_H_ 2.26) to C-5/C-7 (δ_C_ 47.2)/C-8 and from H-13 (δ_H_ 1.23) to C-7/C-11(δ_C_ 42.1)/C-12(δ_C_ 178.9), indicated the fragment of C-3–C-4(C-15)–C-5–C-6–C-7–C-11(C-13)–C-12. By comprehensive analysis of the ^1^H-^1^H COSY spectrum and HMBC correlations (Figure 2), the guaiane skeleton of **2** was deduced. The ^13^C-NMR signals at δ_C_ 72.4 and 73.8, both of which were quaternary carbons, indicated that the epoxide ring exists between C-1 (δ_C_ 73.8) and C-5 (δ_C_ 72.4). The guaiane skeleton, carbonyl group and the epoxide ring accounted for 4 degrees of unsaturation, which suggested the presence of one another ring. The methine signal at δ_C_ 80.7 (C-8) and the carbonyl at δ_C_ 178.9 (C-12), combined with the HMBC correlations, were possible to deduce the occurrence of a lactone ring. Thus the planar structure of compound **2** was established as shown (Figure 2). The ROESY experiment (Figure 4) showed correlations of H-8 (δ_H_ 3.89) to Me-14 (δ_H_ 1.21, β-oriented), and from H-7 (δ_H_ 2.01) to H-8 and Me-13 (δ_H_ 1.23), suggested that H-7, H-8, Me-13 and Me-14 were β-oriented. The correlations of H-11 (δ_H_ 2.29) to H-6a (δ_H_ 1.87) and H-6a to Me-15 (δ_H_ 0.95) indicated that H-6a, H-11 and Me-15 were α-oriented. The stereochemistry of the epoxide ring between C-1 and C-5 of **2,** was assigned to be β-oriented based on the absence of ROESY correlations between H-4 or Me-15 and H-8, which method has been used for the similar known compound (+)-1,5-epoxy-nor-ketoguaiene isolated from agarwood of *Aquilaria* genus [12]. Based on biosynthetic considerations, the stereogenic center C-4 was proposed to be *S*, and consequently, the absolute configuration of compound **2** was assumed to be 1*S*, 4*S*, 5*S*, 7*R*, 8*R*, 10*S*, and 11*R*.

The structure of compound 1,5,9-trimethyl-1,5,9-cyclododecatriene (**3**), which was isolated from agarwood for the first time, was deduced by analyzing the MS, 1D and 2D NMR spectra. The 1D and 2D NMR data (Table 1) of **3** were also firstly reported in this study. The structures of compounds **4**–**9**, which have been isolated from agarwood, were identified by comparison of their spectroscopic data with those reported in the literatures as 4-*epi*-15-hydroxyacorenone (**4**) [13], 7αH-9(10)-ene-11,12-epoxy-8-oxoeremophilane (**5**) [7], neopetasane (**6**) [14], (1β,4αβ,7β,8αβ)-octahydro-7-[1-(hydroxymethyl)ethenyl]-1,8α-dimethylnaphthalen-4α(2H)-ol (**7**) [14], valerianol (**8**) [15], 11-hydroxy-valenc-1(l0)-en-2-one (**9**) [16], respectively. Of them, compounds **7** and **9** were isolated from Chinese agarwood of *A. sinensis* for the first time. Among these nine isolated sesquiterpenoids, compound **5** possesses a delicate sweet smell.

These sesquiterpenoids above were evaluated for the inhibitory activity against AChE, and compounds **2**–**9** were assessed for antibacterial activities against *S. aureus* and *R. solanacearum*. The results showed that compounds **1**, **2**, **4**–**6** and the EtOAc extract exhibited different levels of inhibitory activity with inhibition rate arranged from 13.3% to 70.7% (tacrine as the positive control; inhibition rate: 73.3%). Compounds **4**, **5** and **8** showed antibacterial activities against both of the two strains, and compound **6** was inhibitory towards *R. solanacearum* (kanamycin sulfate as the positive control).

From this paper, sesquiterpenes **1** and **2** belong to the guaiane type, and compounds **5**–**9** were all sesquiterpenes with eremophilane skeleton, while **3** and **4** were also sesquiterpenes but not the main type in agarwood. In addition, eleven eudesmane sesquiterpenes were also reported from our previous phytochemical study on this material [17]. Compared with the wild agarwood, the main types of sesquiterpenoids of Chinese agarwood induced by artificial holing are quite similar [6].

## 3. Materials and Methods

### 3.1. General Imformation

The IR spectra (KBr pellets) were run on a 380 FT-IR instrument from Nicolet (Thermo, Pittsburgh, PA, USA). The HRMS were recorded with an API QSTAR Pulsar mass spectrometer (Bruker, Bremen, Germany). The UV spectra were obtained from a DU-800 spectrometer (Beckman, Brea, CA, USA). Optical rotations were measured on an Autopol III polarimeter (Rudolph, Hackettstown, NJ, USA). CD spectra were recorded with a J-815 spectrometer (JASCO, Tokyo, Japan). The NMR spectra were recorded on an AV-500 spectrometer (500 MHz for ^1^H-NMR and 125 MHz for ^13^C-NMR; Bruker), using the solvent residue signal as the internal standard. Column chromatography was performed with ODS gel (20–45 mm, Fuji Silysia Chemical Co. Ltd., Durham, NC, USA), Sephadex LH-20 (Merck, Darmstadt, Germany) and silica gel (60–80, 200–300 mesh, Qingdao Haiyang Chemical Co. Ltd., Qingdao, China). TLC was carried out on silica gel G precoated plates (Qingdao Haiyang Chemical Co. Ltd.), and spots were detected by spraying with 5% H_2_SO_4_ in EtOH followed by heating. 

### 3.2. Plant Material

Chinese agarwood induced by artificial holing from *A. sinensis* were collected from Xishuangbanna, Yunnan province, P.R. China, in November 2012. The botanical identification was made by Associate Prof. Jun Wang, Institute of Tropical Bioscience and Biotechnology, Chinese Academy of Tropical Agricultural Sciences, where a voucher specimen (No. 20121108) was deposited.

### 3.3. Extraction and Isolation

Dried powdered Chinese agarwood (4.7 Kg) induced by artificial holing was refluxed with 95% EtOH (5 L × 6). The EtOH extract (510.0 g) was suspended in H_2_O (2.5 L) and partitioned with EtOAc (2.5 L × 3), and then *n*-BuOH (2.5 L × 3). The EtOAc extract (310.0 g) was applied to silica gel vacuum liquid chromatography with a step gradient elution of CHCl_3_–MeOH (*v*/*v* 1:0 to 0:1) to provide nine fractions (Fr.1–Fr.9). Fr.1 (143.2 g) was subjected to silica gel CC and eluted with PE–Me_2_CO step gradient (*v*/*v* 1:0 to 0:1) to get nine fractions (Fr.1-1–Fr.1-9). Fr.1-1 (3.8 g) was applied to ODS gel eluting with MeOH−H_2_O step gradient (*v*/*v* 3:7 to 1:0), then submitted to repeated CC on silica gel eluting with PE–CHCl_3_ (*v*/*v* 6:4 to 10:9) and Sephadex LH-20 (CHCl_3_–MeOH 1:1) to obtained compounds **2** (3.0 mg), **3** (2.0 mg), **5** (16.0 mg), **6** (18.0 mg), and **8** (62.0 mg). Fr.1-4 (16.8 g) was purified using ODS gel with a step gradient elution of MeOH−H_2_O (*v*/*v* 3:7 to 1:0), followed by silica gel CC eluting with CHCl_3_−MeOH (*v*/*v* 200:1) to obtain compound **4** (4.5 mg). Fr.1-5 (15.8 g) was submitted to column chromatography over silica gel eluted with CHCl_3_–MeOH (*v*/*v* 200:1 to 100:1), and further purification with Sephadex LH-20 (CHCl_3_–MeOH 1:1) to yield compounds **7** (102.0 mg) and **9** (32.0 mg). Compound **1** (4.0 mg) from Fr.1-8 was chromatographed on ODS gel eluting with MeOH−H_2_O (*v*/*v* 3:7 to 1:0) and then applied to silica gel and eluted with CHCl_3_–MeOH (*v*/*v* 40:1).

*3-Oxo-7-hydroxylholosericin A* (**1**). Colorless oil; C_15_H_20_O_5_; [α${]}_{\text{D}}^{24}$ + 104.0 (*c* 0.50, MeOH); UV (MeOH) λ_max_ (log ε): 272 (4.78), 254 (4.68); CD (*c* 0.1, MeOH) λ_max_ (∆ε): 204 (−10.18), 298 (+1.68) nm; IR (KBr) υ_max_ 3434, 2921, 1630, 1597, 1384, 1115, 709 cm^−1^; ^1^H NMR (CD_3_OD, 500 MHz), ^1^H-NMR (DMSO, 500 MHz) and ^13^C-NMR (CD_3_OD, 125 MHz) data, see Table 1; HREIMS *m*/*z*: [M + CF_3_COO]^−^ 393.1166 (calcd. 280.1311 for C_15_H_20_O_5_).

*1,5;8,12-Diepoxyguaia-12-one* (**2**). Colorless oil; C_15_H_22_O_3_; [α${]}_{\text{D}}^{24}$ − 113.0 (*c* 0.75, MeOH); UV (MeOH) λ_max_ (log ε): 199 (4.12) nm; IR (KBr) υ_max_ 3448, 2929, 1771, 1712, 1456, 1172, 988 cm^−1^; ^1^H-NMR (CDCl_3_, 500 MHz) and ^13^C-NMR (CDCl_3_, 125 MHz) data, see Table 1; HREIMS *m*/*z*: 250.1563 [M]^+^ (calcd for C_15_H_22_O_3_, 250.1569).

### 3.4. AChE Inhibition Activity

These above isolated compounds were tested for their acetylcholinesterase inhibitory activities with the spectrophotometric method developed by Ellman [18] with slightly modification. Acetylcholinesterase, 5,5′-dithio-bis-(2-nitrobenzoic) acid (DTNB, Ellman’s reagent), and *S-*acetylthiocholine iodide were purchased from Sigma Chemical (Saint Louis, MO, USA). The detail experimental procedures were the same as those published previously [8]. The inhibition rates were calculated as follows: % inhibition = (E − S)/E × 100 (E is the activity of the enzyme without test compound, and S is the activity of enzyme with test compounds). The values are expressed as the mean ± SD of triplicate experiments. The AChE inhibitory activity experiment results are shown in Table 2.

### 3.5. Antibacterial Activity

Compounds **2**–**9** were evaluated *in vitro* for their antibacterial activities against *S. aureus* and *R. solanacearum* by the filter paper disc agar diffusion method respectively [19]. The two strains were maintained on a nutrient agar (NA) slant at 4 °C. The isolated compounds dissolved in MeOH (50 μL, 10 mg/mL) respectively were impregnated on sterile filter paper discs (6 mm diameter) and then applied aseptically to the surface of the agar plates. Kanamycin sulfate (50.0 μL, 0.50 mg/mL) was used as positive control, and MeOH (50.0 μL) was used as negative control. The plates were incubated at 37 °C for 24 h. The diameters of the inhibition zones including the 6 mm disc diameter were measured. Experiments were carried out in triplicate and the results are expressed as the mean value. The results of the antibacterial activities test were in Table 3.

## 4. Conclusions

Phytochemical investigations on the EtOAc extract of Chinese agarwood induced by artificial holing led to the isolation of nine sesquiterpenoids, including two new guaiane skeletons, namely 3-oxo-7-hydroxylholosericin A (**1**) and 1,5;8,12-diepoxy-guaia-12-one (**2**). All the isolates and the EtOAc extract were assessed for the inhibitory activity against AChE and compounds **2**–**9** were tested for antibacterial activities against *S. aureus* and *R. solanacearum*. The results showed that compounds **1**, **2, 4**–**6** and the EtOAc extract exhibited different levels of inhibitory activity with inhibition rate arranged from 13.3% to 70.7% at one concentration 50 µg/mL. Compounds **4**, **5** and **8** showed antibacterial activities at the same concentration of 0.50 mg/mL against both of the two strains, and compound **6** was inhibitory towards *R. solanacearum*.

## Figures and Tables

**Table 1:** ^1^H (500 MHz) and ^13^C (125 MHz) NMR spectral data of compounds **1**–**3** (δ in ppm, *J* in Hz).

No.	1 ^c^			2 ^b,c^		3 ^b,c^	
	δ_C_^a^	δ_H_^a^	δ_H_ (in DMSO)	δ_C_	δ_H_	δ_C_	δ_H_
1	89.5			73.8		135.4	
2	45.4	2.42 d (18.5), 2.17 d (18.5)	2.40 d (18.5), 2.05 d (18.5)	29.6	2.04 m, 1.74 m	125.2	5.12 t (6.6)
3	219.0			26.4	1.65 m, 1.14 m	26.6	2.04 m
4	48.6	2.36 dq (2.0, 7.3)	2.16 dq (1.6, 7.0)	39.6	2.21 d (1.7)	32.4	2.04 m
5	44.7	2.12 m	2.00 m	72.4		135.4	
6	28.2	2.33 br d (14.8), 2.26 dd (7.3, 14.8 )	2.17 br d (14.1), 2.09 dd (7.4, 14.1)	28.0	2.26 m, 1.87 dd (8.5, 12.3)	125.2	5.12 t (6.6)
7	75.1			47.2	2.01 m	26.6	2.04 m
8	112.9			80.7	3.89 ddd (11.3, 10.2, 3.0)	32.4	2.04 m
9	43.3	2.53 d (13.8), 1.97 d (13.8)	2.39 d (13.4), 1.77 d (13.4)	35.2	1.98 m, 1.93 dd (12.9, 3.9)	135.4	
10	77.1			30.3	2.59 m	125.2	5.12 t (6.6)
11	152.5			42.1	2.29 m	26.6	2.04 m
12	71.3	4.62 dt (12.8, 2.4), 4.42 dt (12.8, 2.4)	4.49 dt (12.6, 2.2), 4.26 dt (12.6, 2.2)	178.9		32.4	2.04 m
13	105.8	5.28 br t (2.4), 5.07 br t (2.4)	5.18 br t (2.2), 4.97 br t (2.2)	12.8	1.23 d (7.0)	23.6	1.68 s
14	28.6	1.35 s	1.23 s	17.0	1.21 d (7.3)	23.6	1.68 s
15	12.4	1.06 d (7.3)	0.96 d (7.0)	16.8	0.95 d (7.2)	23.6	1.68 s
7-OH			5.16 s				
10-OH			5.00 s				

^a^ Measured in CD_3_OD, ^b^ Measured in CDCl_3_, ^c^ Chemical shifts are given in ppm; *J* values are in parentheses and reported in Hz.

**Table 2:** AChE inhibition activity of the EtOAc extract and compounds **1**–**9** at 50 µg/mL.

Compound	Percentage of Inhibition	Compound	Percentage of Inhibition
**1**	21.1 ± 0.8	**6**	70.7 ± 0.6
**2**	13.3 ± 0.9	**7–9**	<10
**3**	<10	EtOAc	18.5 ± 0.9
**4**	14.7 ± 0.9	Tacrine ^a^	73.3 ± 0.8
**5**	17.8 ± 0.6		

^a^ Positive control.

**Table 3:** Antibacterial activity of compounds **2**–**9** against two strains (mm).

Compound	*S. aureus*	*R. solanacearum*
**2, 3**	–	–
**4**	12.35 ± 0.21	16.90 ± 0.09
**5**	9.87 ± 0.14	9.02 ± 0.25
**6**	–	8.07 ± 0.16
**7**	–	–
**8**	10.10 ± 0.12	8.86 ± 0.13
**9**	–	–
Kanamycin sulfate ^a^	24.06 ± 0.29	30.64 ± 0.13

Notes: ^a^ Positive control; “–“ inactive; each value represents the mean ± SD (*n =* 3).

## Supplementary Materials

Supplementary materials can be accessed at: http://www.mdpi.com/1420-3049/21/3/274/s1.
