# Supplementary material for: Sesquiterpenoids from Chinese Agarwood Induced by Artificial Holing
Source: Molecules. 2016 Feb 26;21(3):274. doi: 10.3390/molecules21030274 (PMC6273283; doi:10.3390/molecules21030274)
Supplement: Supplementary file 1 [file molecules-21-00274-s001.pdf]

Supplementary Materials: Sesquiterpenoids from Chinese Agarwood Induced by Artificial Holing

Wei Li, Ge Liao, Wen-Hua Dong, Fan-Dong Kong, Pei Wang, Hao Wang, Wen-Li Mei and Hao-Fu Dai

S-1. Theory and Calculation Details.

Figure S1. ^{1}H-NMR spectrum (500 MHz) of compound **1** in CD_3OD.
Figure S2. ^{13}C-NMR spectrum (125 MHz) of compound **1** in CD_3OD.
Figure S3. ^{1}H-^{1}H COSY spectrum (500 MHz) of compound **1** in CD_3OD.
Figure S4. HSQC spectrum (500 MHz) of compound **1** in CD_3OD.
Figure S5. HMBC spectrum (500 MHz) of compound **1** in CD_3OD.
Figure S6. ROESY spectrum (500 MHz) of compound **1** in CD_3OD.
Figure S7. ^{1}H-NMR spectrum (500 MHz) of compound **1** in DMSO.
Figure S8. ^{13}C-NMR spectrum (125 MHz) of compound **1** in DMSO.
Figure S9. ^{1}H-^{1}H COSY spectrum (500 MHz) of compound **1** in DMSO.
Figure S10. HSQC spectrum (500 MHz) of compound **1** in DMSO.
Figure S11. HMBC spectrum (500 MHz) of compound **1** in DMSO.
Figure S12. ROESY spectrum (500 MHz) of compound **1** in DMSO.
Figure S13. NOE difference experiment irradiated at H-4 of compound **1**.
Figure S14. ESI(+)MS spectrum of compound **1**.
Figure S15. HRESI(+)MS spectrum of compound **1**.

Figure S16. ^{1}H-NMR spectrum (500 MHz) of compound **2** in $CDCl_3$.
Figure S17. ^{13}C-NMR spectrum (125 MHz) of compound **2** in $CDCl_3$.
Figure S18. ^{1}H-^{1}H COSY spectrum (500 MHz) of compound **2** in $CDCl_3$.
Figure S19. HSQC spectrum (500 MHz) of compound **2** in $CDCl_3$.
Figure S20. HMBC spectrum (500 MHz) of compound **2** in $CDCl_3$.
Figure S21. ROESY spectrum (500 MHz) of compound **2** in $CDCl_3$.
Figure S22. ESI(+)MS spectrum of compound **2**.
Figure S23. HRESI(+)MS spectrum of compound **2**.

Figure S24. ^{1}H-NMR spectrum (500 MHz) of compound **3** in $CDCl_3$.
Figure S25. ^{13}C-NMR spectrum (125 MHz) of compound **3** in $CDCl_3$.
Figure S26. ^{1}H-^{1}H COSY spectrum (500 MHz) of compound **3** in $CDCl_3$.
Figure S27. HSQC spectrum (500 MHz) of compound **3** in $CDCl_3$.
Figure S28. HMBC spectrum (500 MHz) of compound **3** in $CDCl_3$.
Figure S29. ROESY spectrum (500 MHz) of compound **3** in $CDCl_3$.

S-1. Theory and Calculation Details.

The calculations were performed by using the density functional theory (DFT) as carried out in the Gaussian 03 [1]. The preliminary conformational distributions search was performed by HyperChem 7.5 software (Hypercube, Inc., Saint-Lambert, Canada). All ground-state geometries were further optimized at the B3LYP/6-31G(d) level. Conformers within a 2 kcal/mol energy threshold from the global minimum were selected to calculate the electronic transitions [2–5]. The overall theoretical ECD spectra were obtained according to the Boltzmann weighting of each conformers. Solvent effects of methanol solution were evaluated at the same DFT level by using the SCRF/PCM method [6–8].

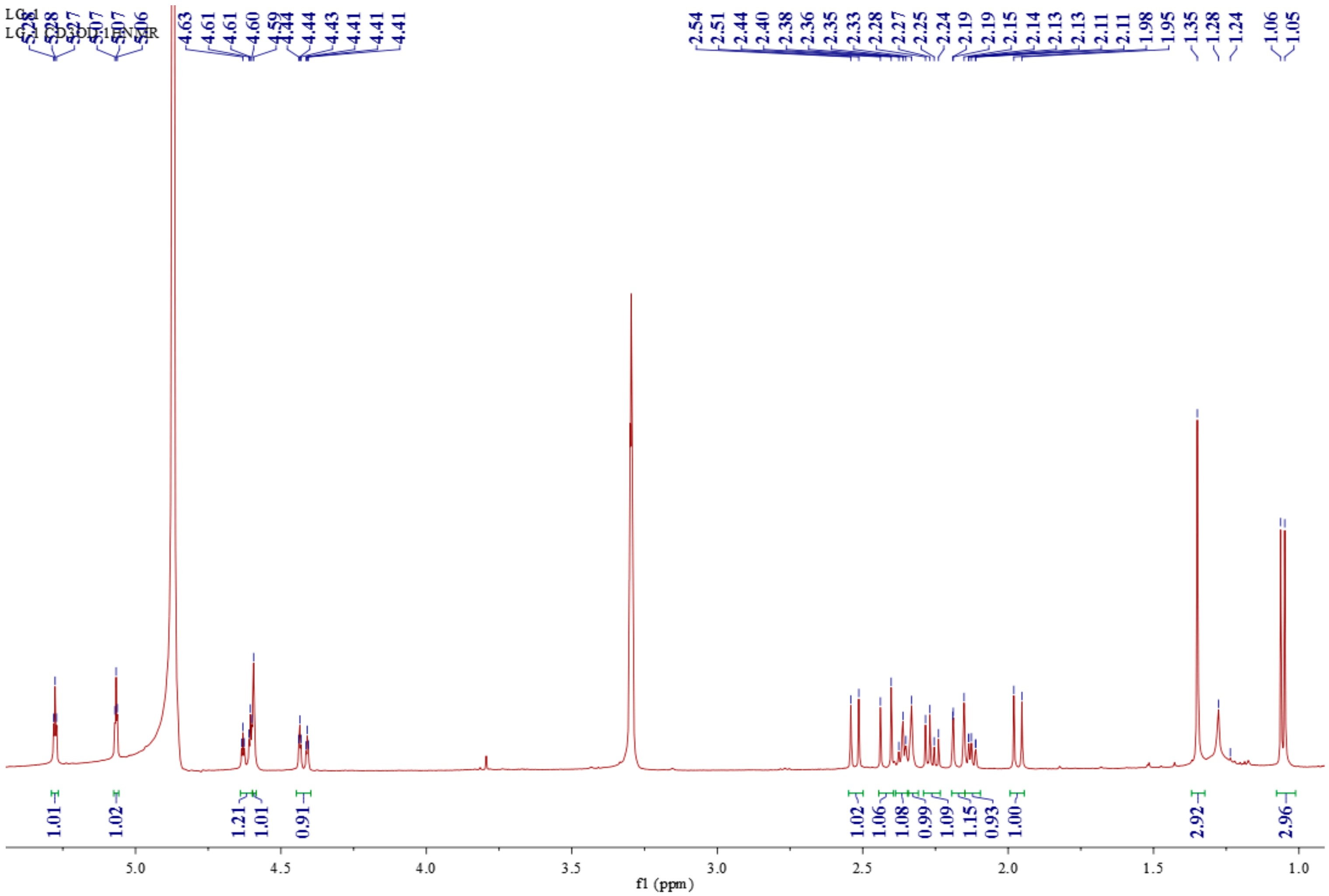

Figure S1. ^{1}H-NMR spectrum (500 MHz) of compound **1** in CD_3OD.

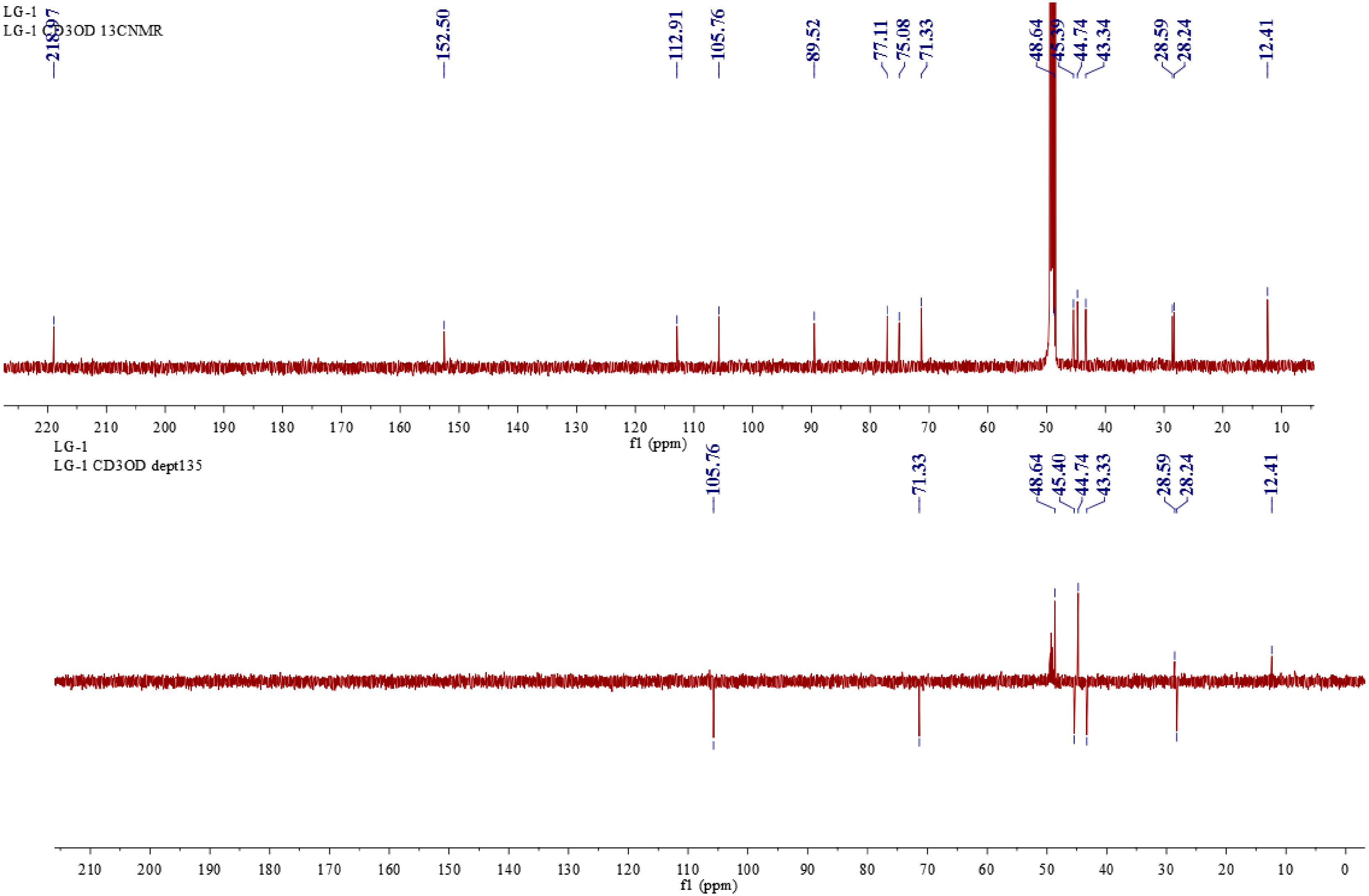

Figure S2. ^{13}C-NMR spectrum (125 MHz) of compound **1** in CD_3OD.

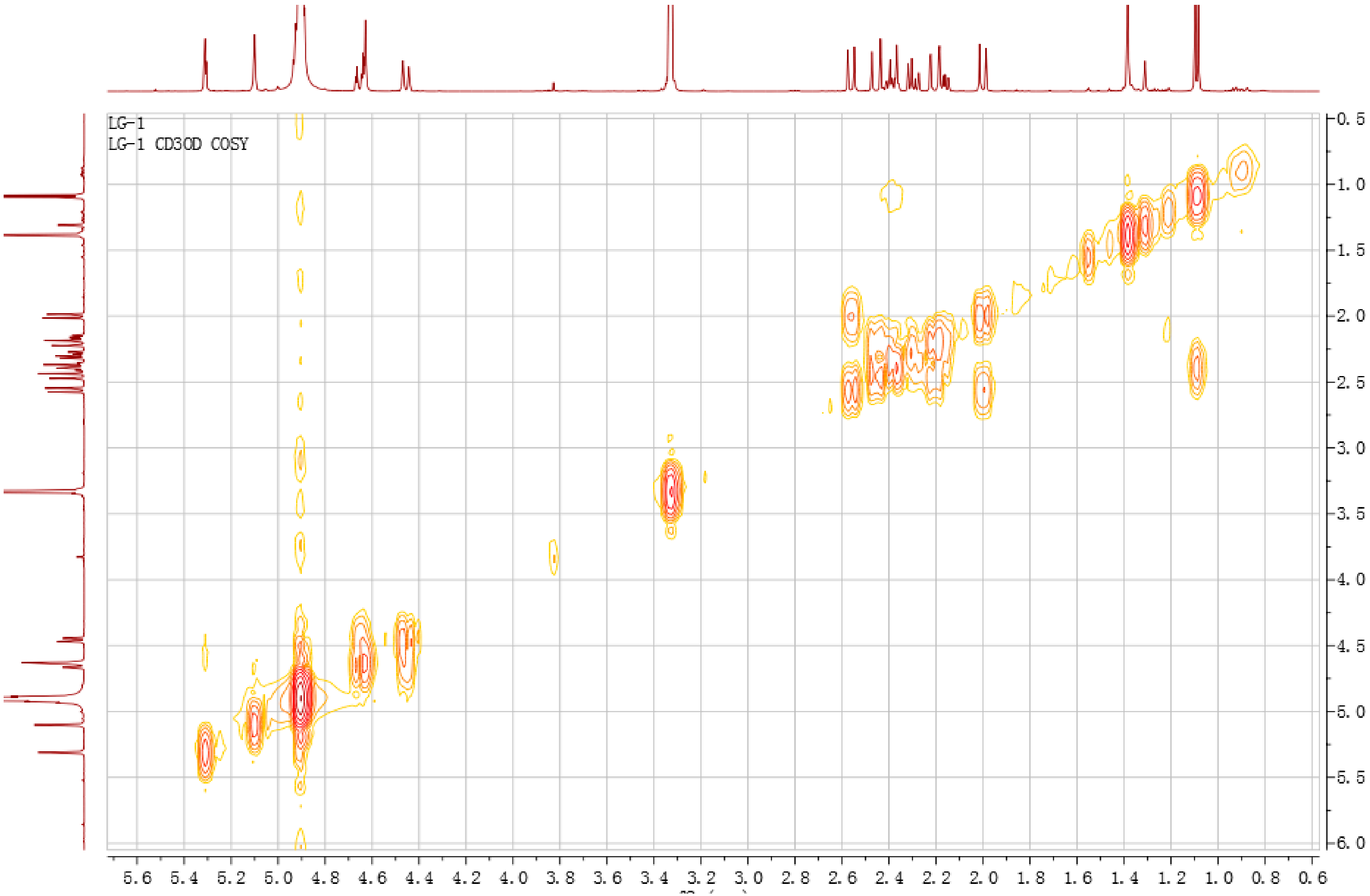

Figure S3. ^{1}H-^{1}H COSY spectrum (500 MHz) of compound **1** in CD_3OD.

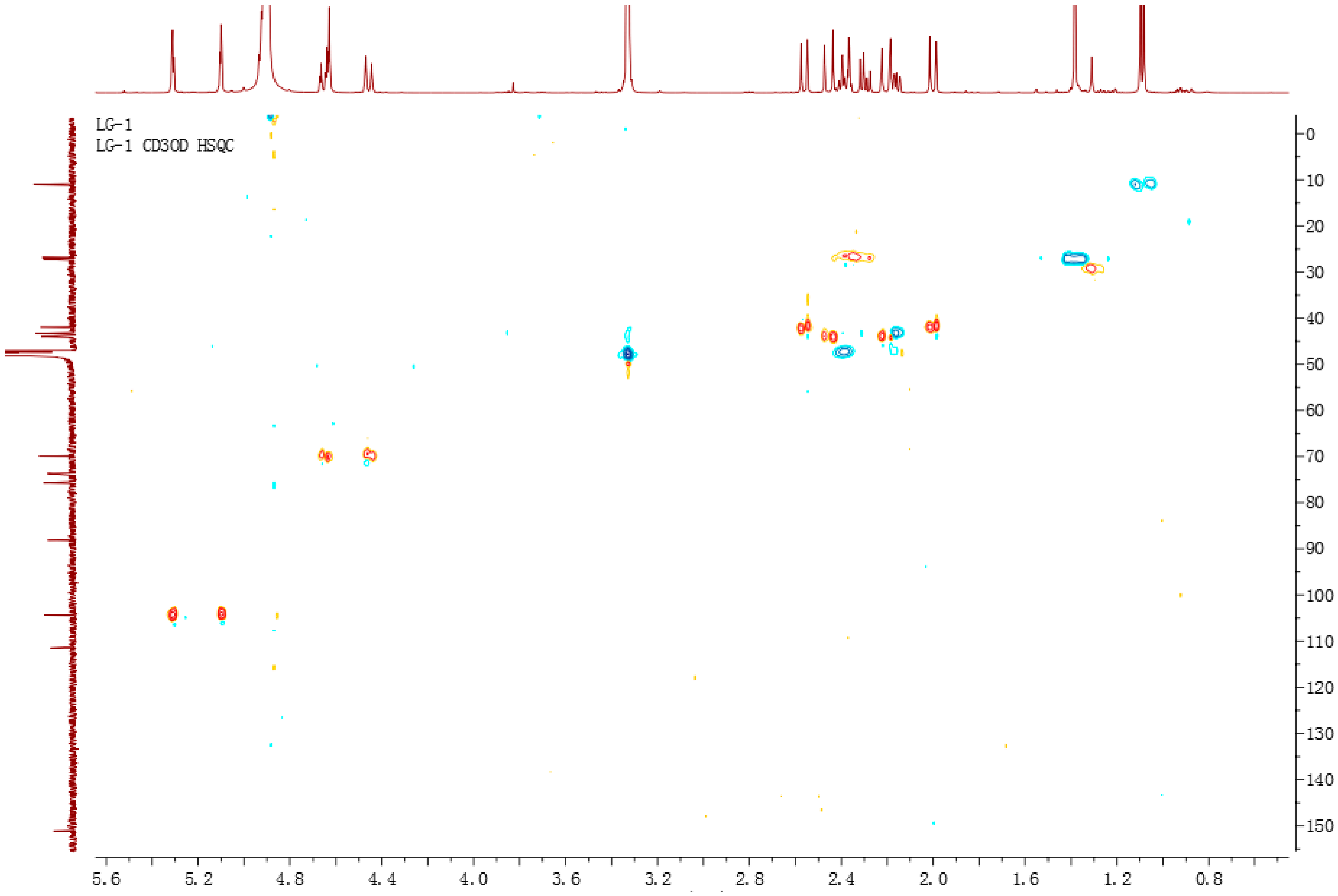

Figure S4. HSQC spectrum (500 MHz) of compound **1** in CD_3OD.

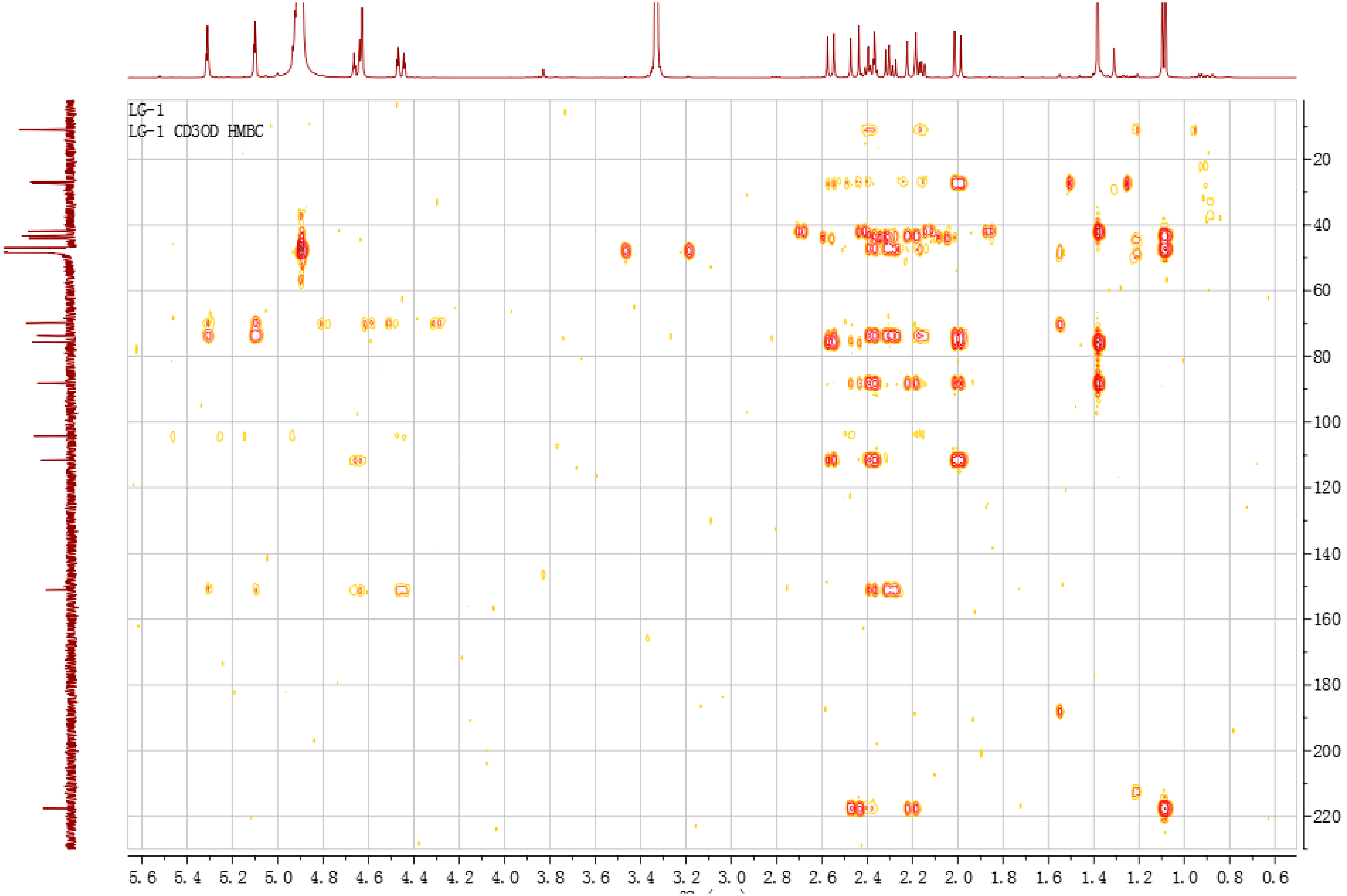

Figure S5. HMBC spectrum (500 MHz) of compound **1** in CD_3OD.

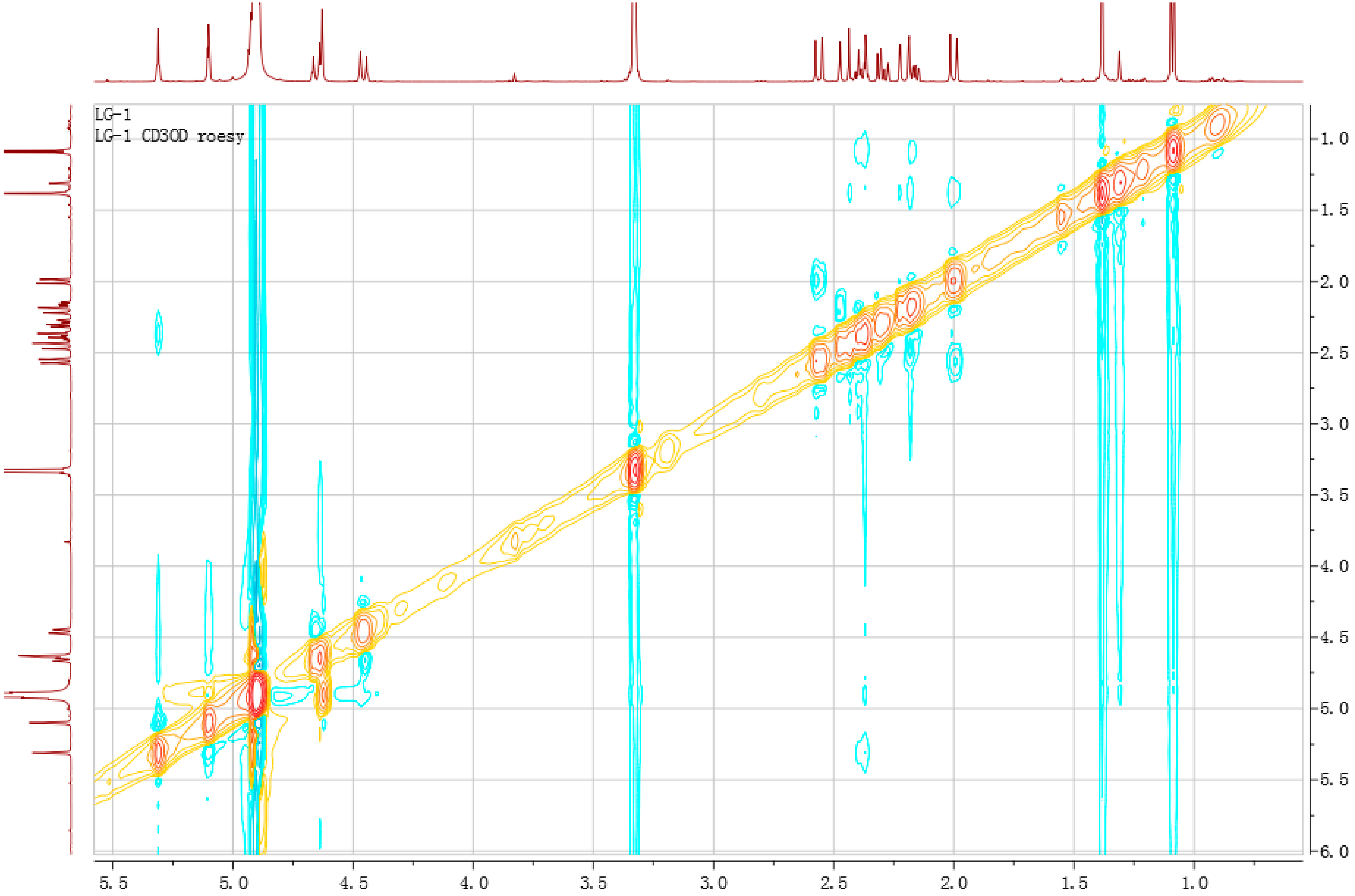

Figure S6. ROESY spectrum (500 MHz) of compound **1** in CD_3OD.

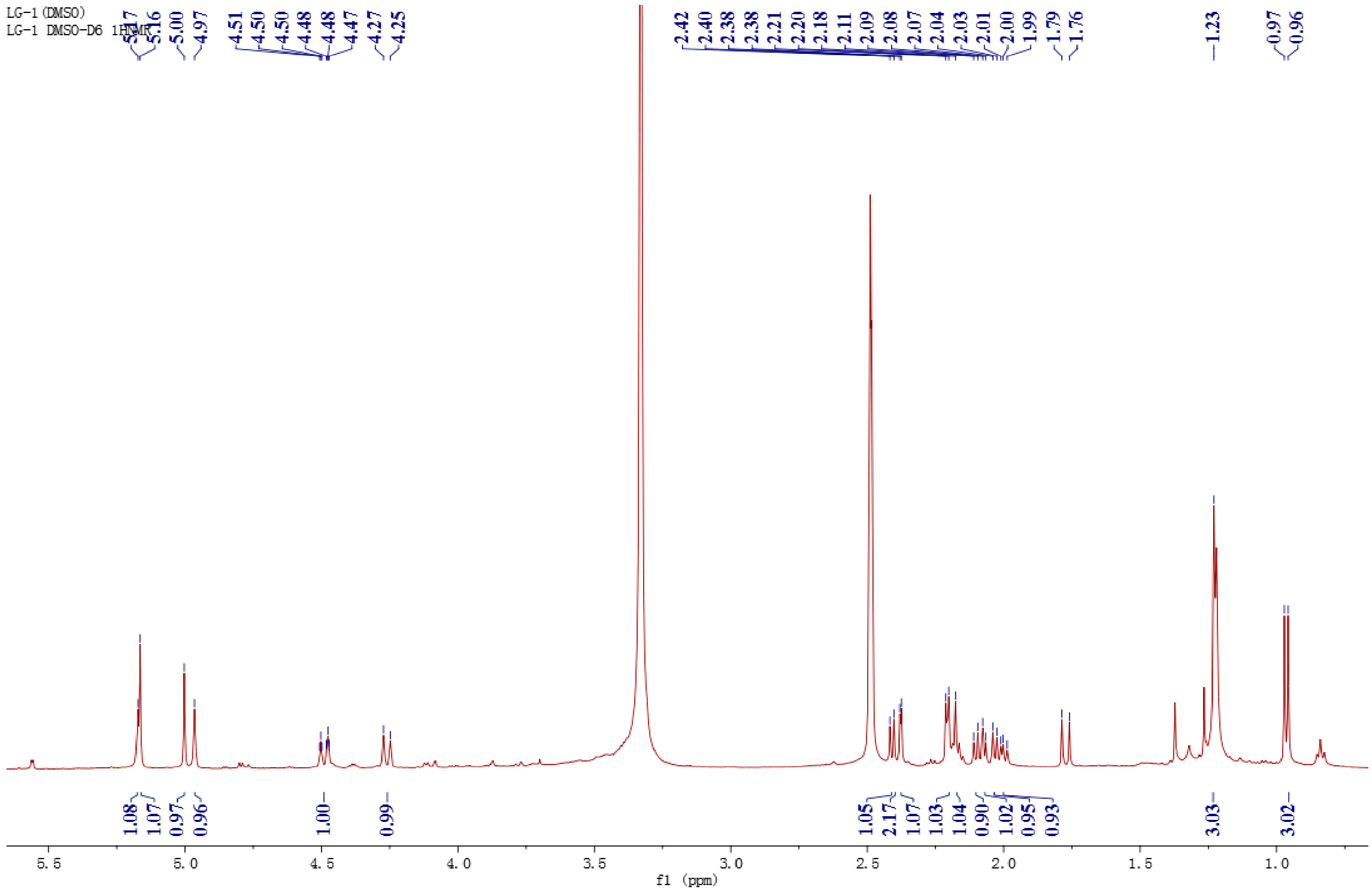

Figure S7. ^{1}H-NMR spectrum (500 MHz) of compound **1** in DMSO.

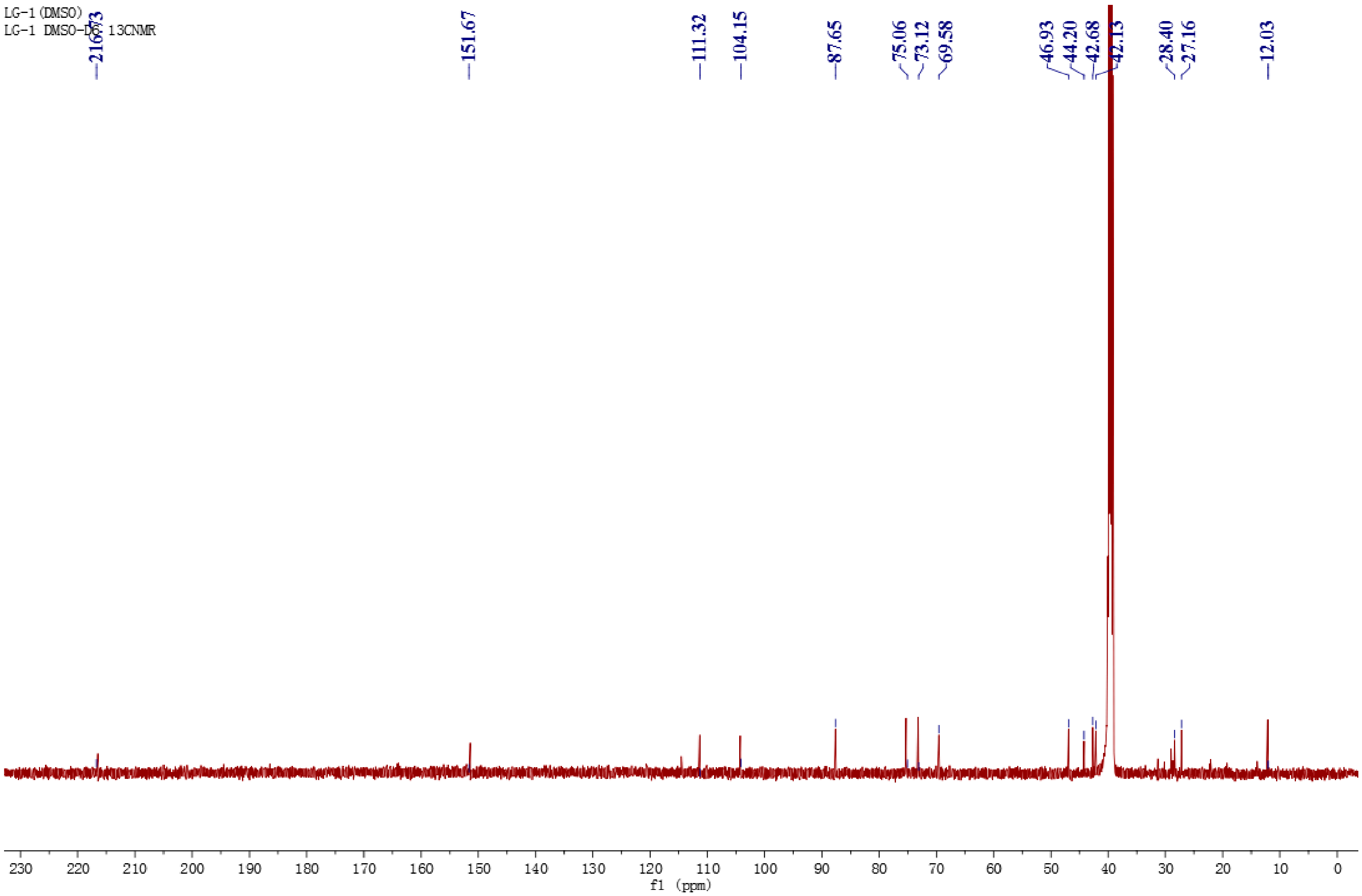

Figure S8. ^{13}C-NMR spectrum (125 MHz) of compound **1** in DMSO.

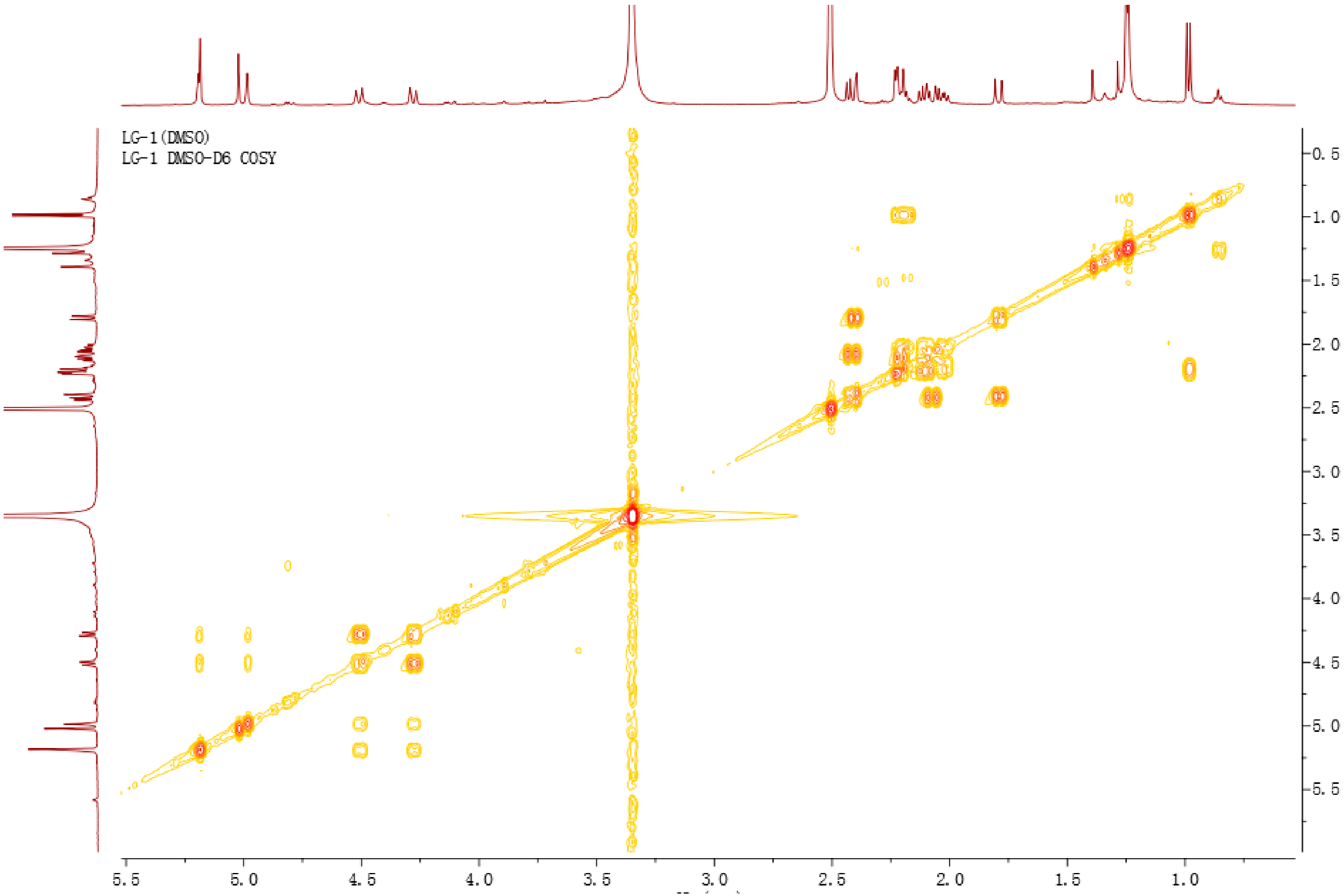

Figure S9. ^{1}H-^{1}H COSY spectrum (500 MHz) of compound **1** in DMSO.

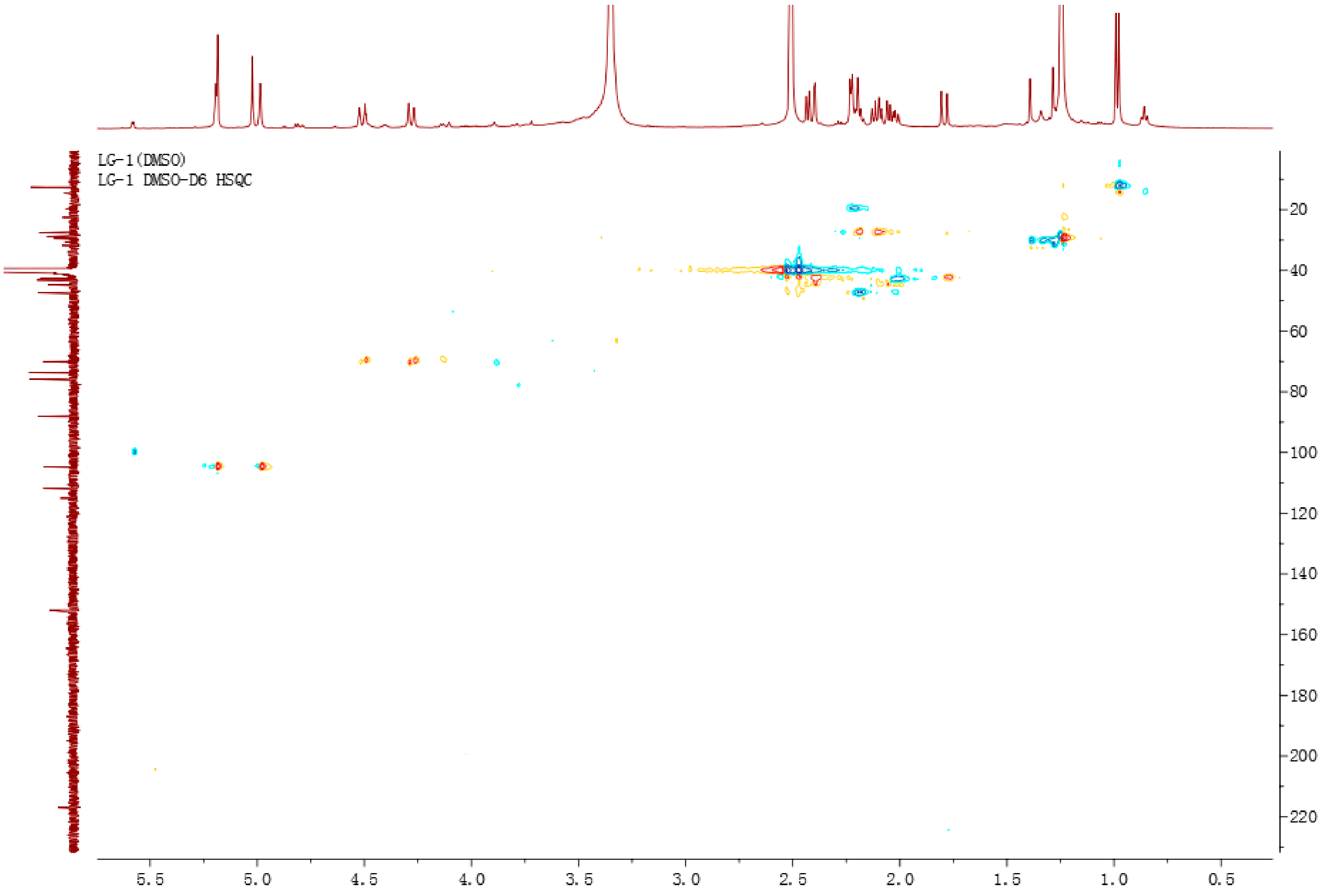

Figure S10. HSQC spectrum (500 MHz) of compound **1** in DMSO.

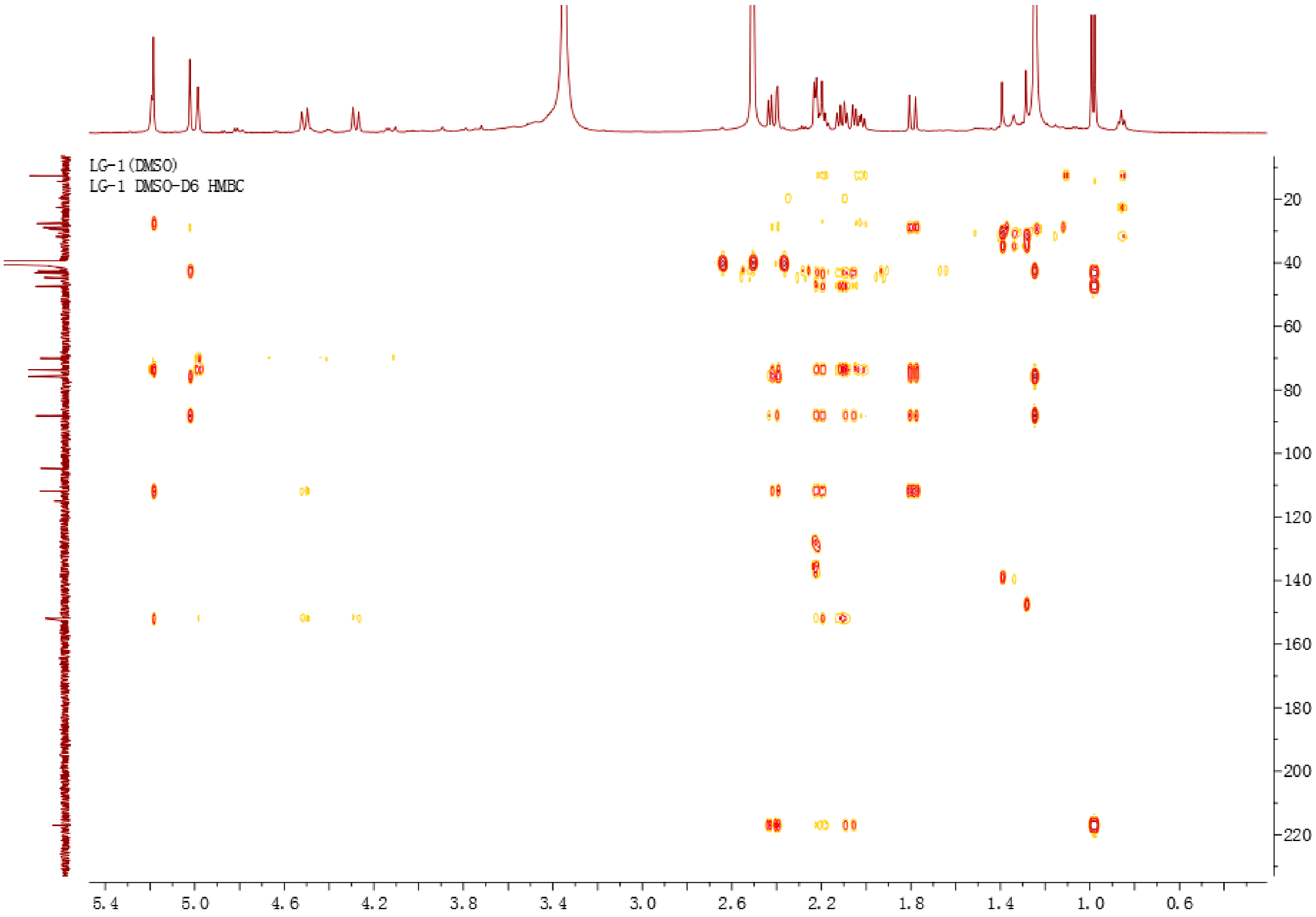

Figure S11. HMBC spectrum (500 MHz) of compound **1** in DMSO.

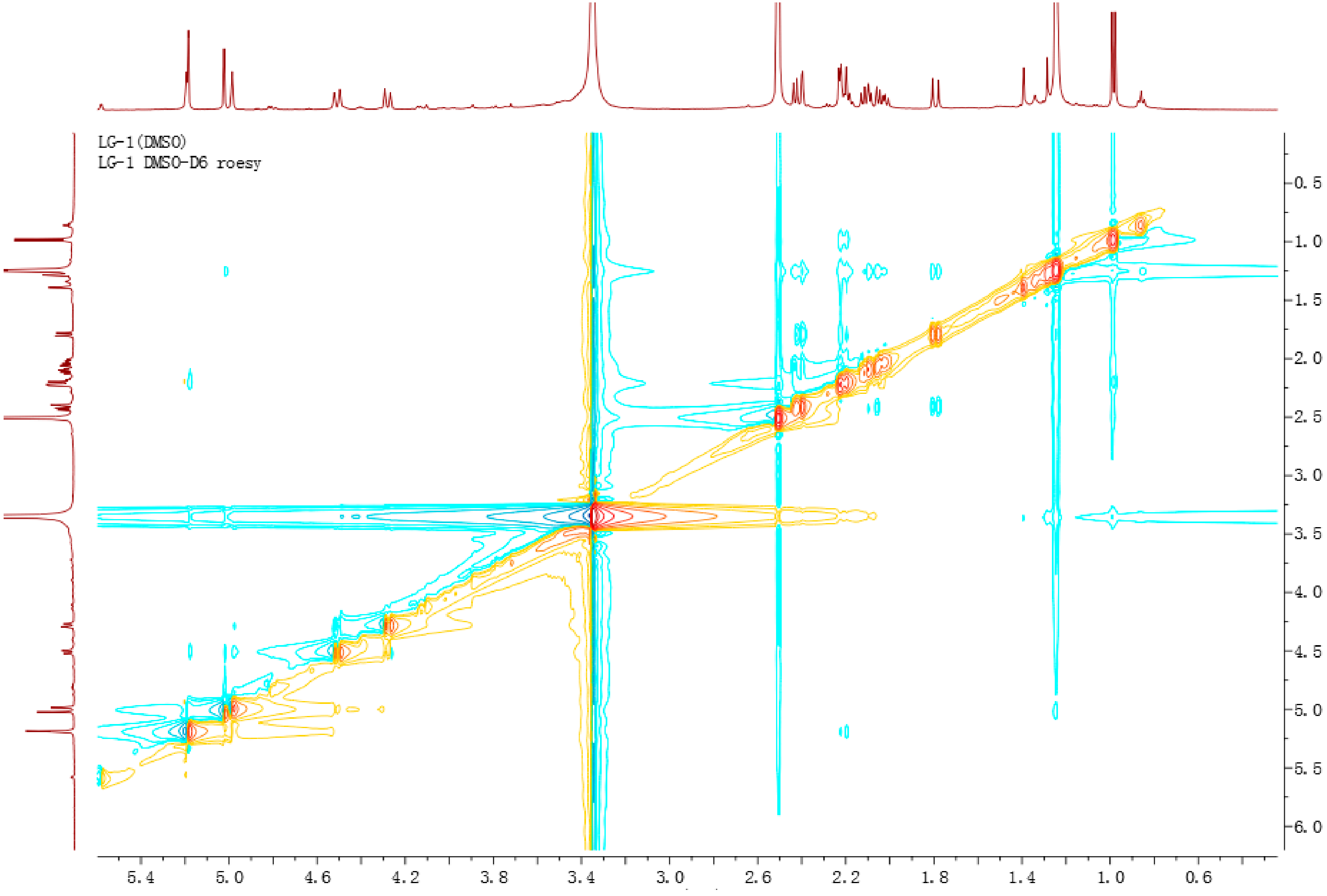

Figure S12. ROESY spectrum (500 MHz) of compound **1** in DMSO.

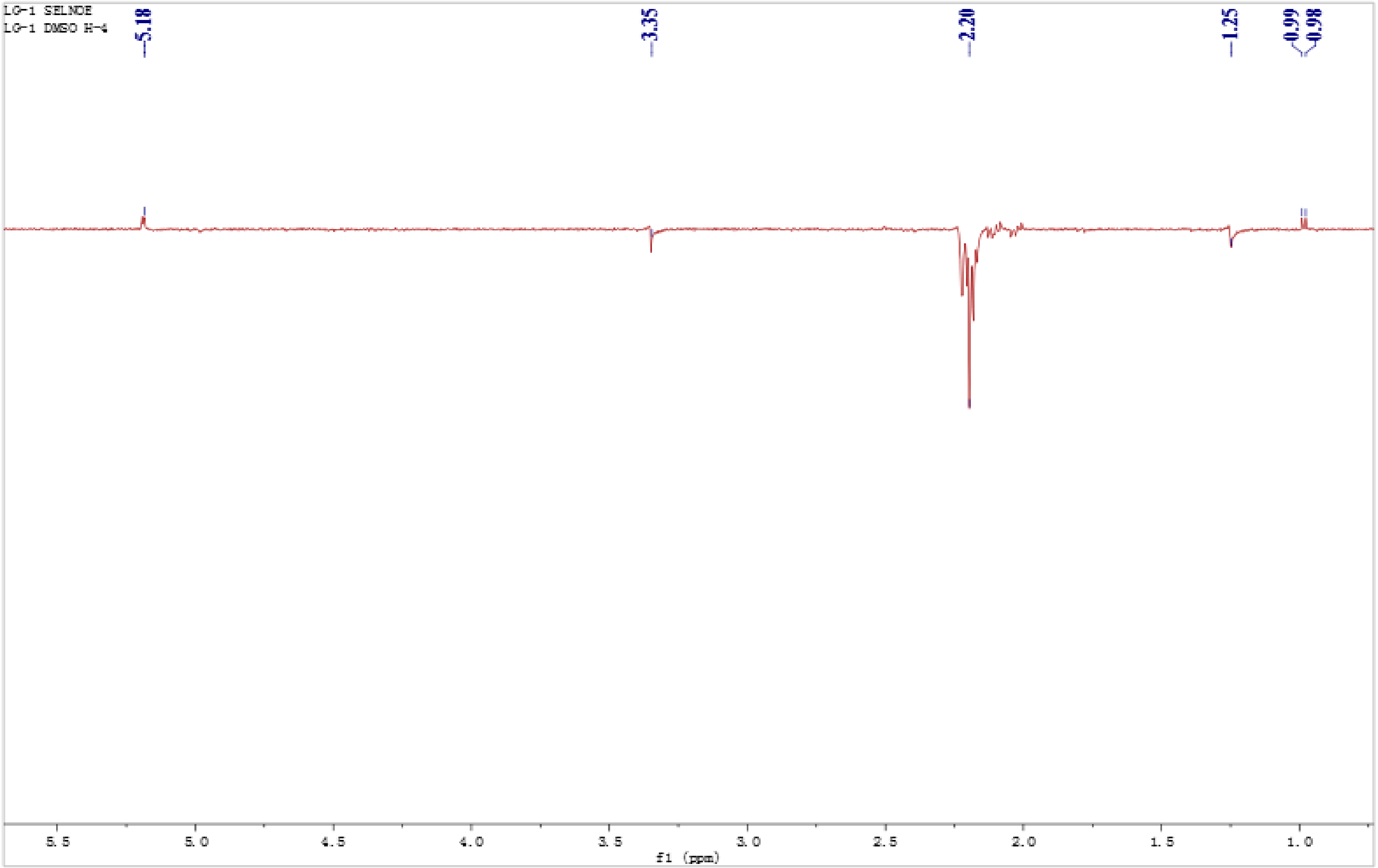

Figure S13. NOE difference experiment irradiated at H-4 of compound **1**.

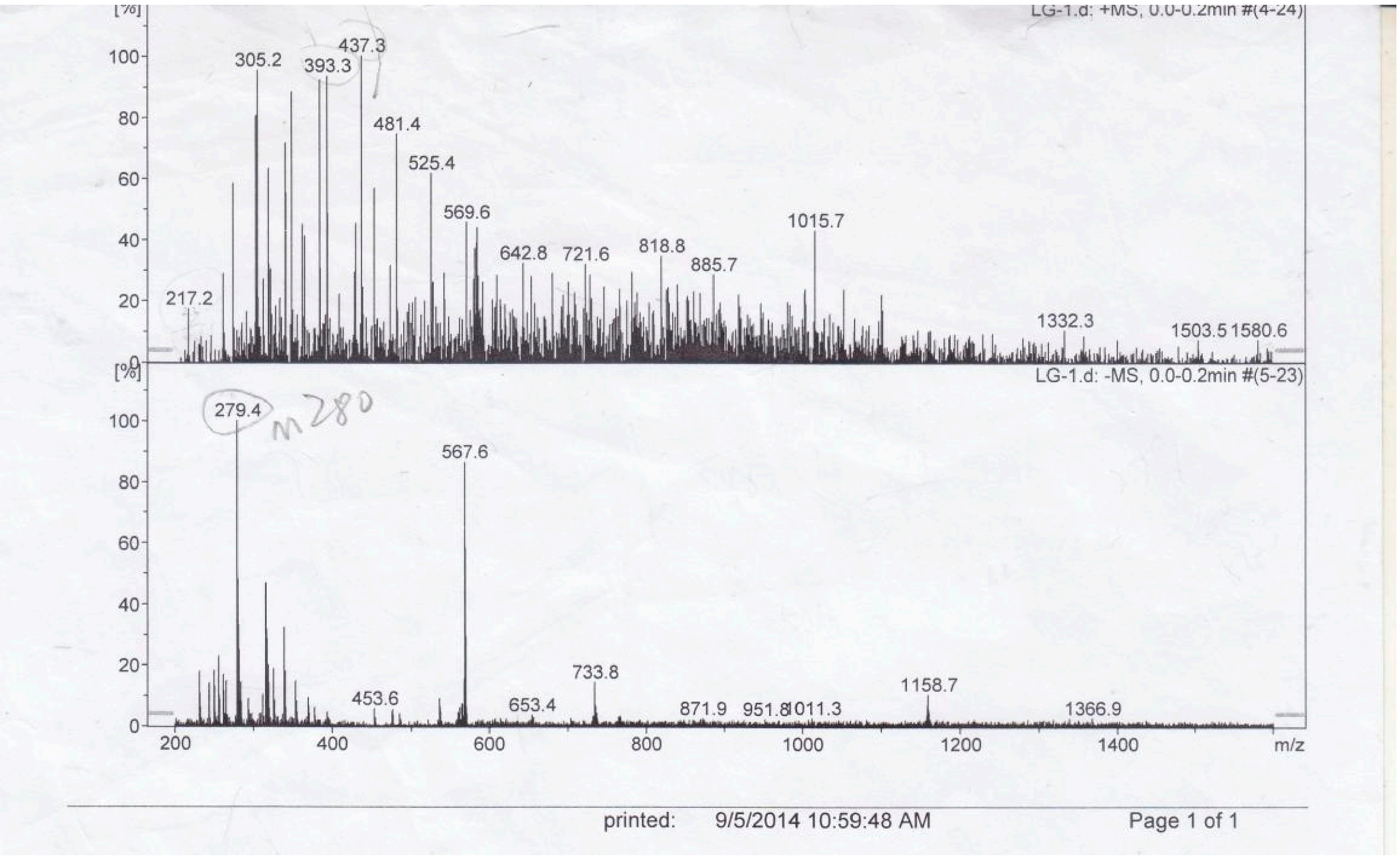

Figure S14. ESI(+)MS spectrum of compound **1**.

Qualitative Analysis Report

**Data Filename**	LG-1.d	**Sample Name**	LG-1
**Sample Type**	Sample	**Position**	P1-C2
**Instrument Name**	Instrument 1	**User Name**	
**Acq Method**	SIBU-ESI-l.m	**Acquired Time**	1/19/2016 1:44:03 PM
**IRM Calibration Status**	Success	**DA Method**	ESI+.m
**Comment**			

**Sample Group**		**Info.**
**Acquisition SW Version**	6200 series TOF/6500 series Q-TOF B.05.01 (B5125.2)	

User Spectra

Fragmentor Voltage	Collision Energy	Ionization Mode
135	0	ESI

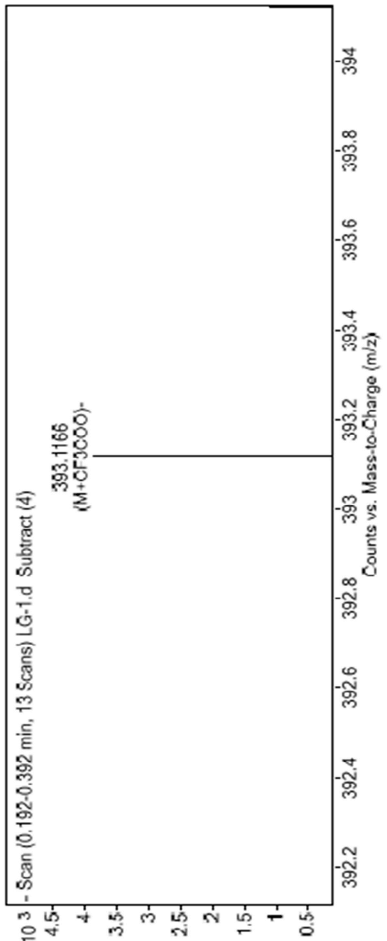

Peak List

m/z	z	Abund	Formula	Ion
255.2332	1	1425.21		

Figure S15. HRESI(+)MS spectrum of compound **1**.

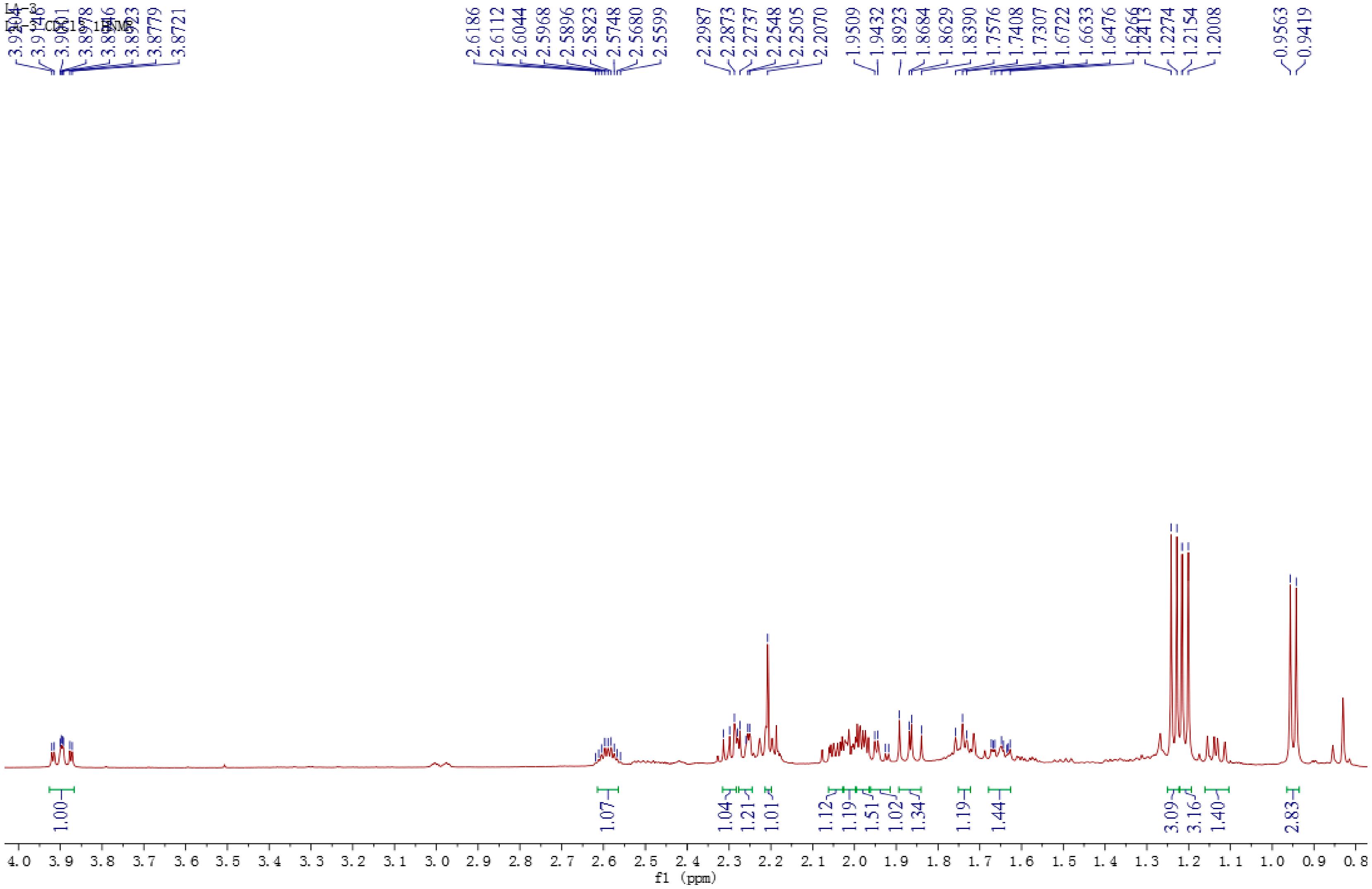

Figure S16. ^{1}H-NMR spectrum (500 MHz) of compound **2** in $CDCl_3$.

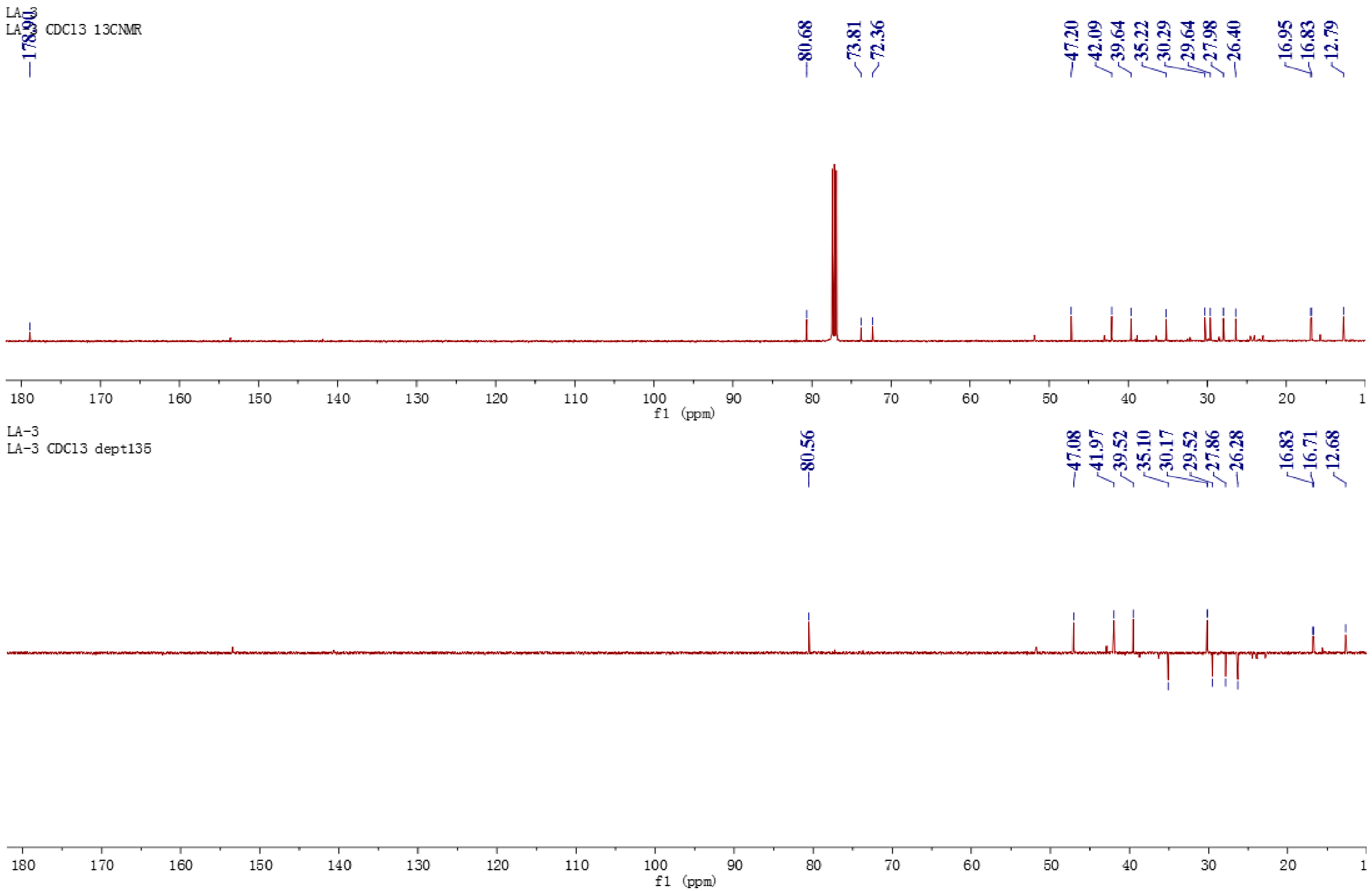

Figure S17. ^{13}C-NMR spectrum (125 MHz) of compound **2** in $CDCl_3$.

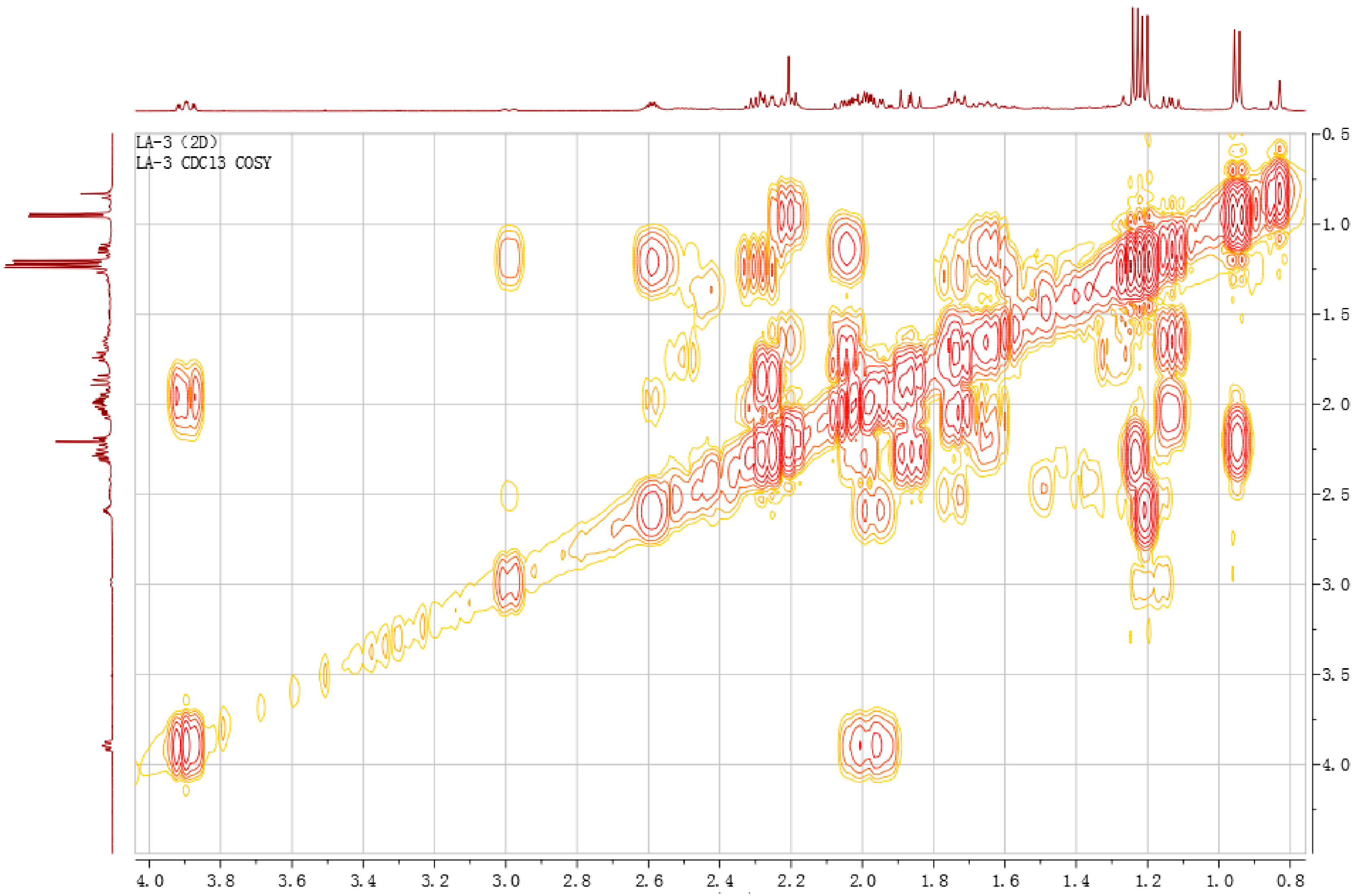

Figure S18. ^{1}H-^{1}H COSY spectrum (500 MHz) of compound **2** in $CDCl_3$.

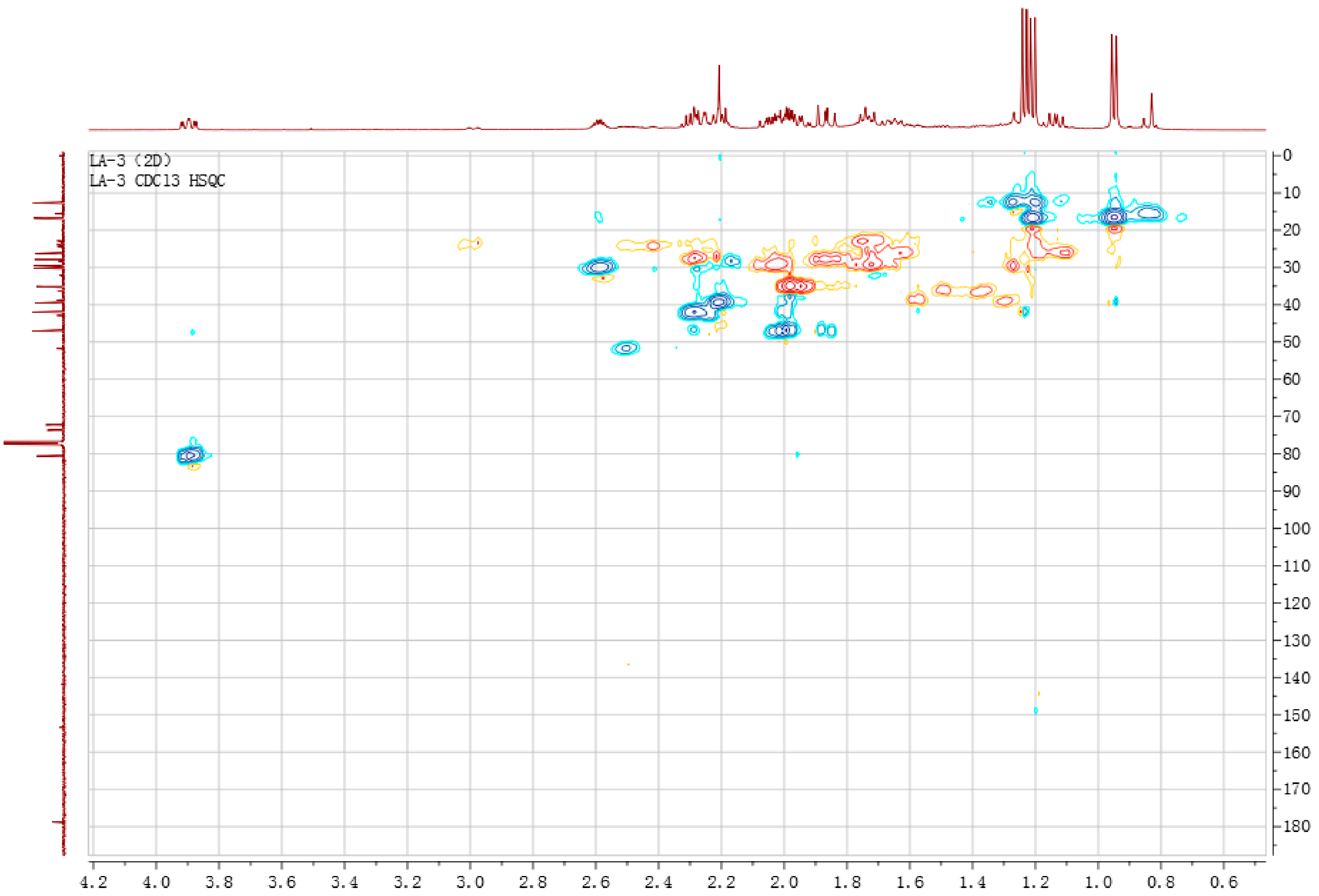

Figure S19. HSQC spectrum (500 MHz) of compound **2** in $CDCl_3$.

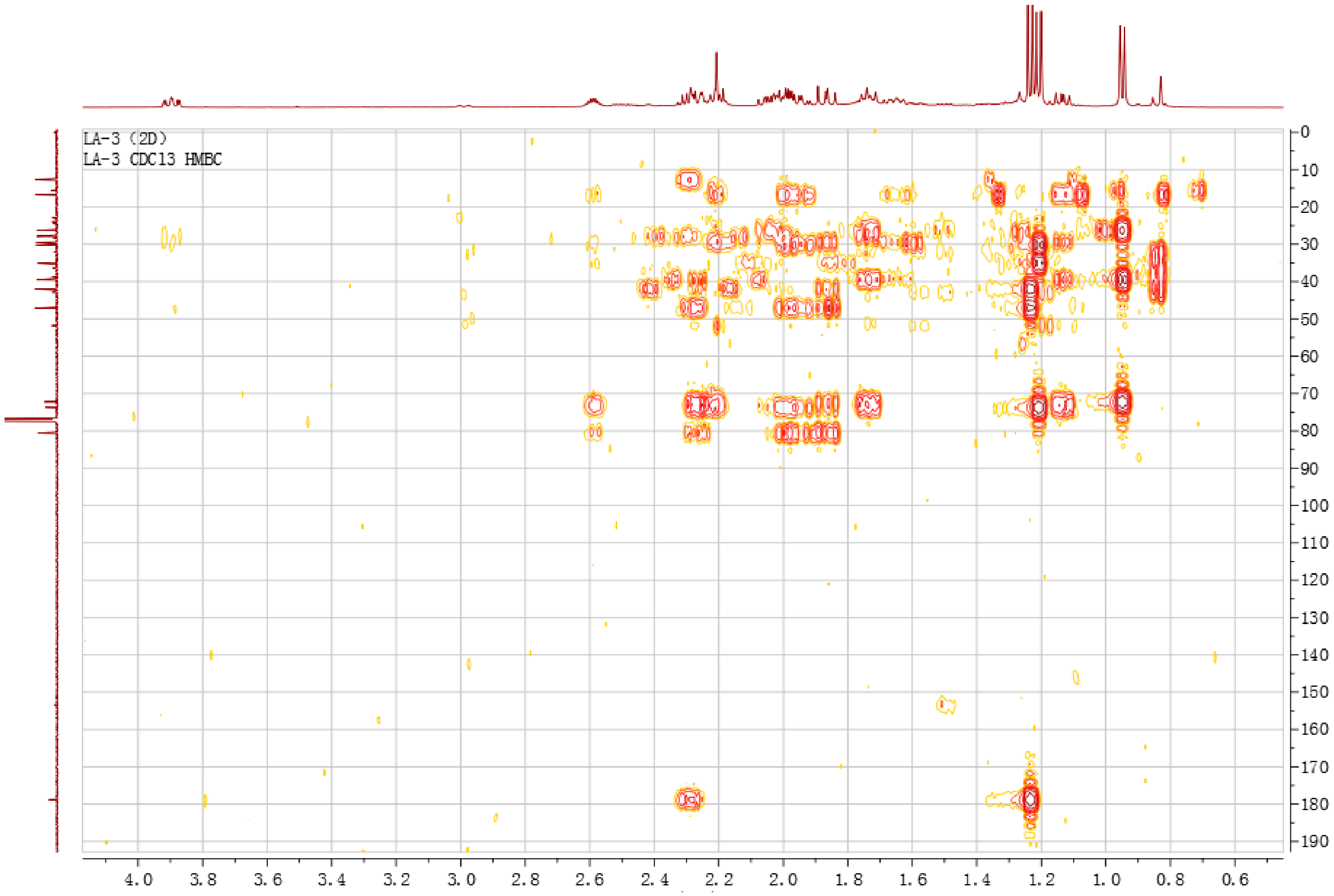

Figure S20. HMBC spectrum (500 MHz) of compound **2** in $CDCl_3$.

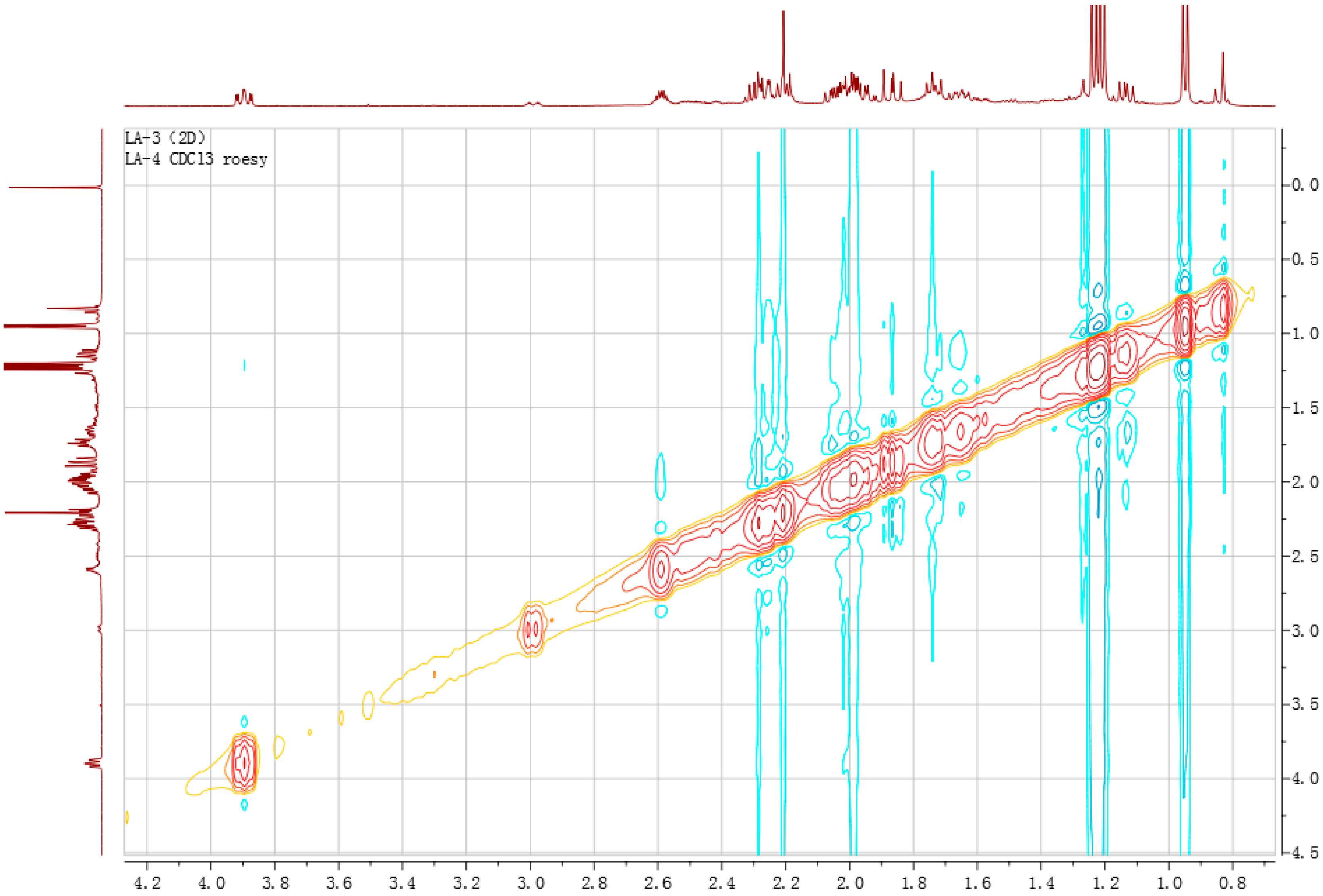

Figure S21. ROESY spectrum (500 MHz) of compound **2** in $CDCl_3$.

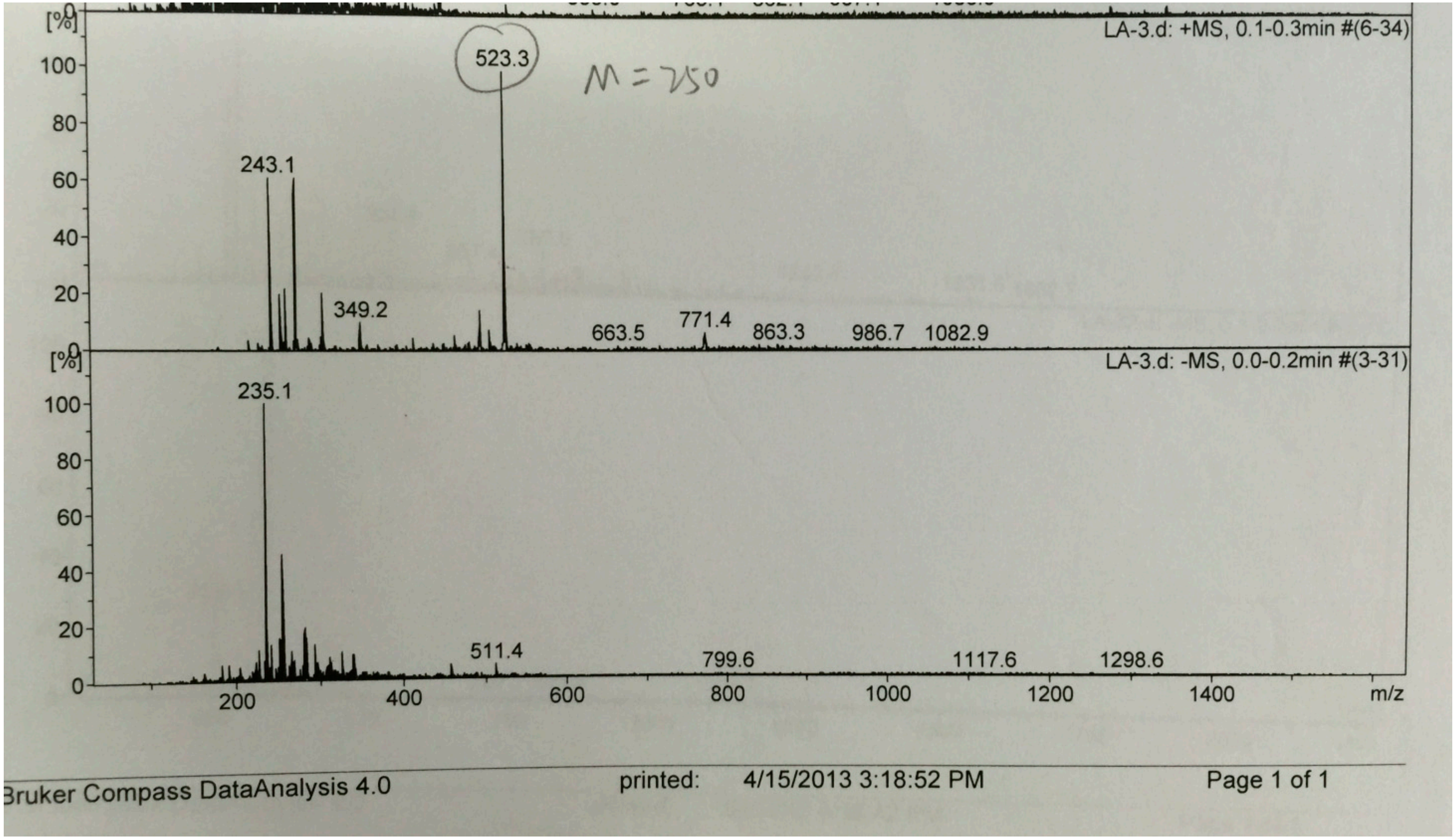

Figure S22. ESI(+)MS spectrum of compound **2**.

Elemental Composition Report

Page 1

Single Mass Analysis

Tolerance = 10.0 PPM / DBE: min = -10.0, max = 120.0

Selected filters: None

Monoisotopic Mass, Odd and Even Electron Ions

13 formula(e) evaluated with 1 results within limits (up to 51 closest results for each mass)

Elements Used:

C: 0-200 H: 0-400 O: 2-4

LA-3 KIB Autospec Premier

11:48:36 24-Oct-2013 M131024EA-12AFAMM 16 (1.470) P776

Voltage EI+ 250.1563 28.1

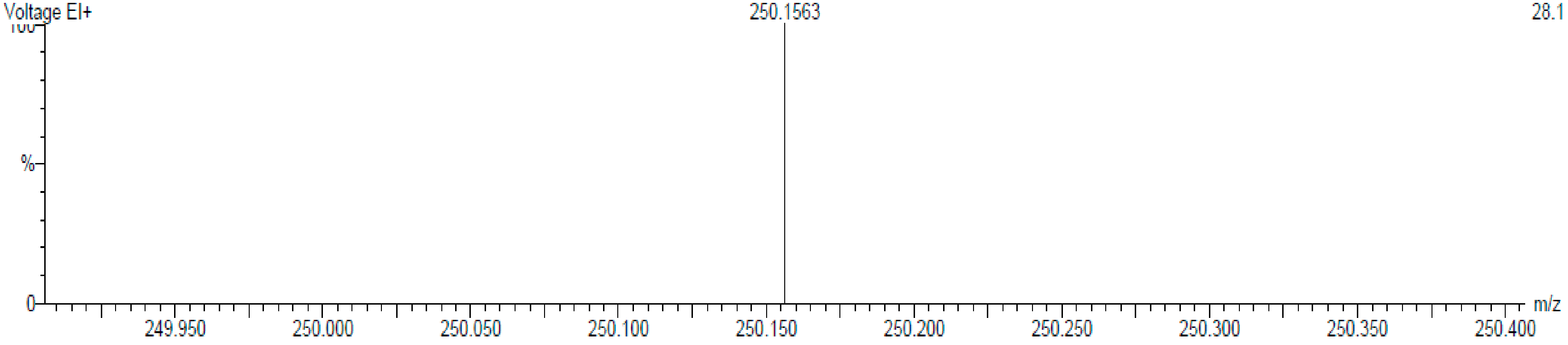

	Mass	Calc. Mass	mDa	PPM	DBE	i-FIT	Formula
Minimum:					-10.0		
Maximum:			200.0	10.0	120.0		
	250.1563	250.1569	-0.6	-2.4	5.0	5546029.0	C15 H22 O3

Figure S23. HRESI(+)MS spectrum of compound **2**.

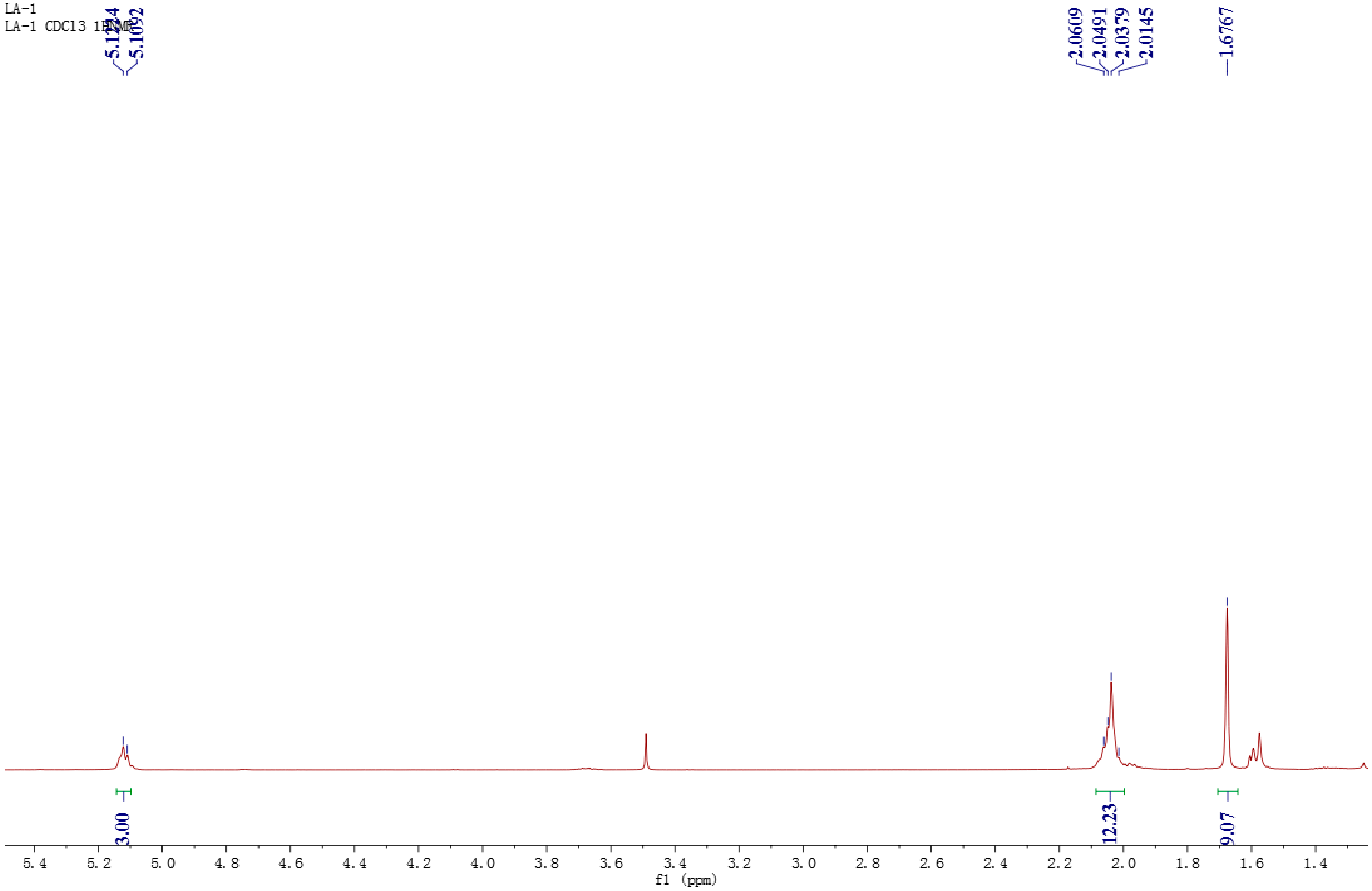

Figure S24. ^{1}H-NMR spectrum (500 MHz) of compound **3** in $CDCl_3$.

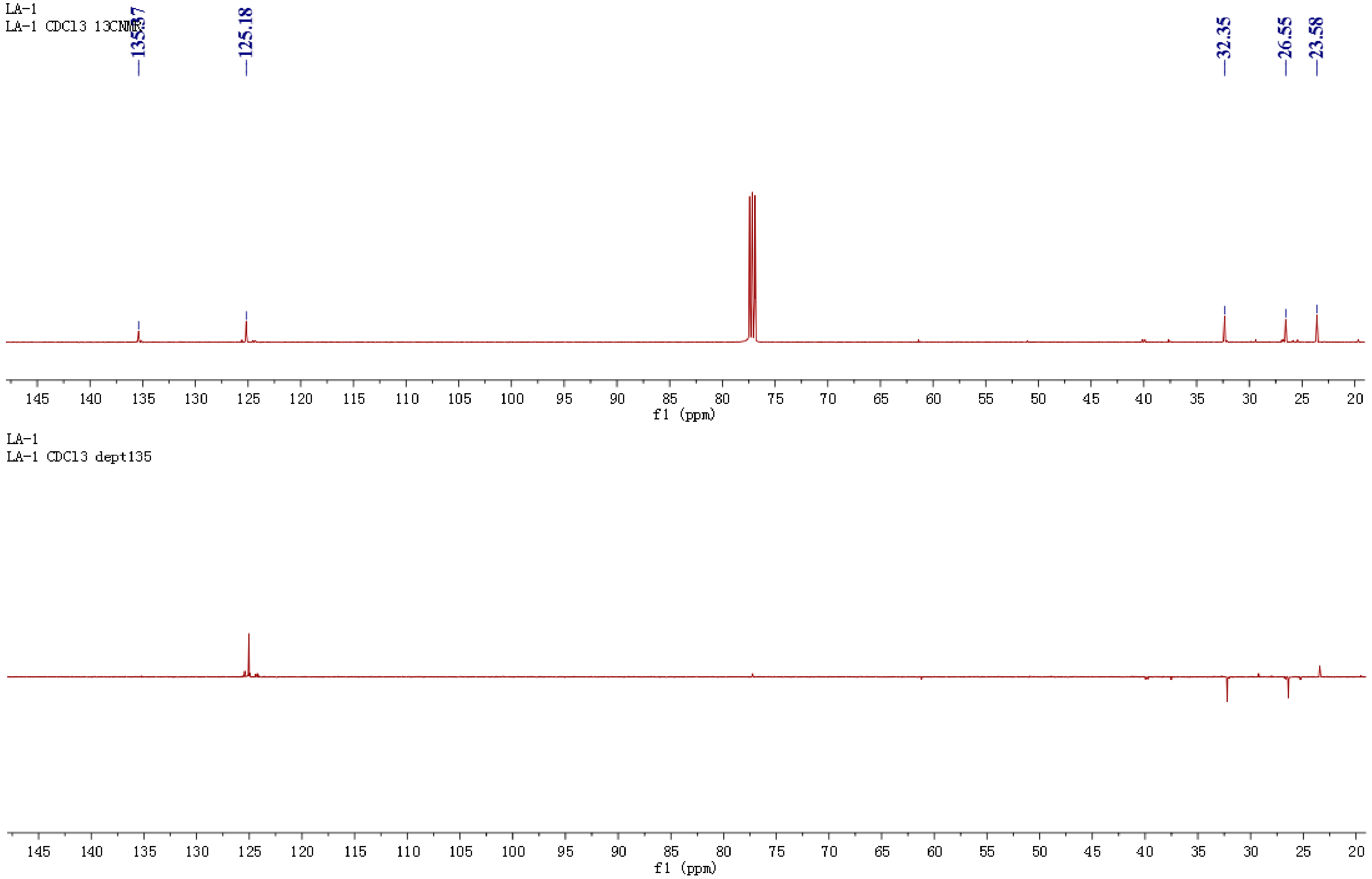

Figure S25. ^{13}C-NMR spectrum (125 MHz) of compound **3** in $CDCl_3$.

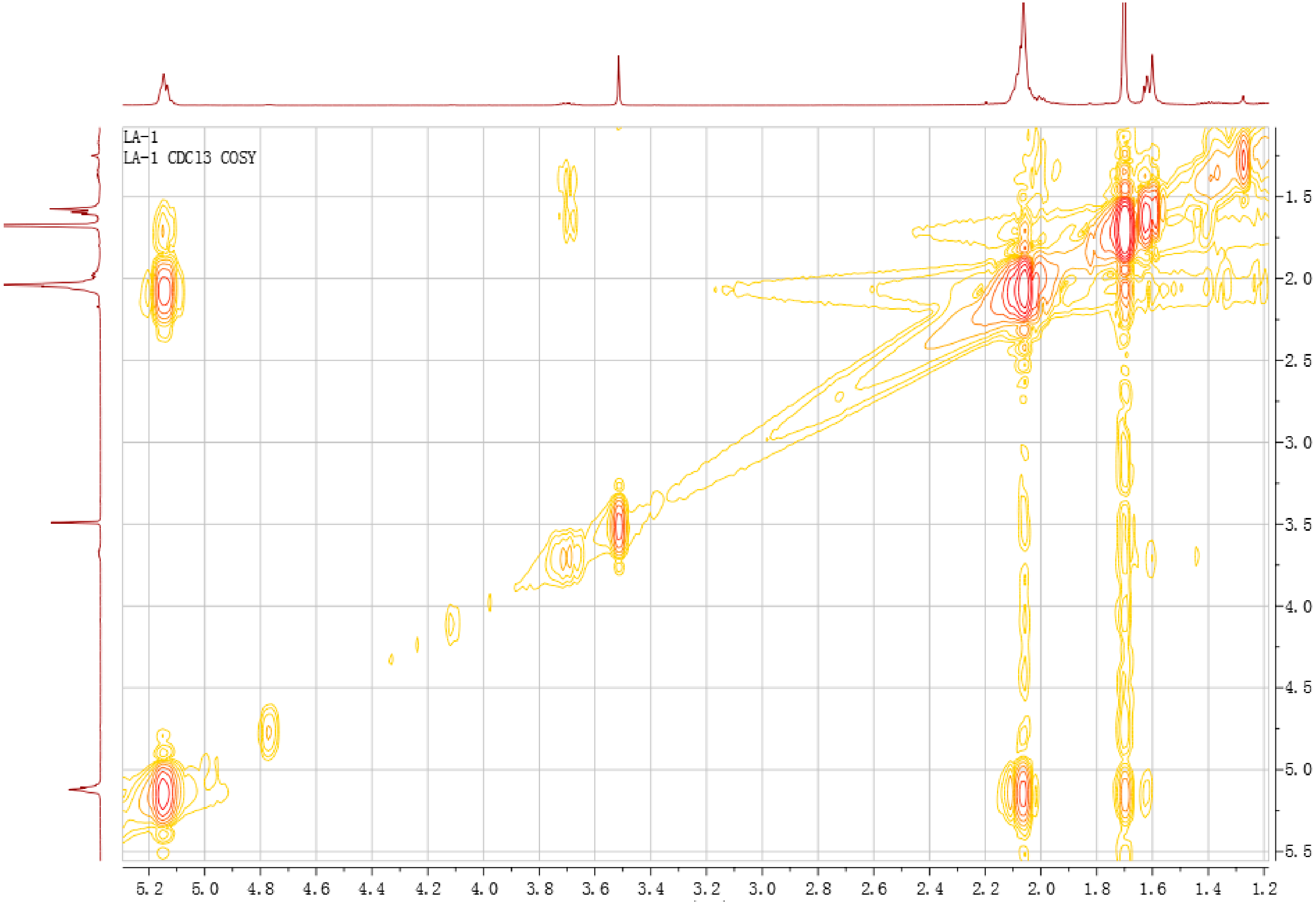

Figure S26. ^1H-^1H COSY spectrum (500 MHz) of compound **3** in $CDCl_3$.

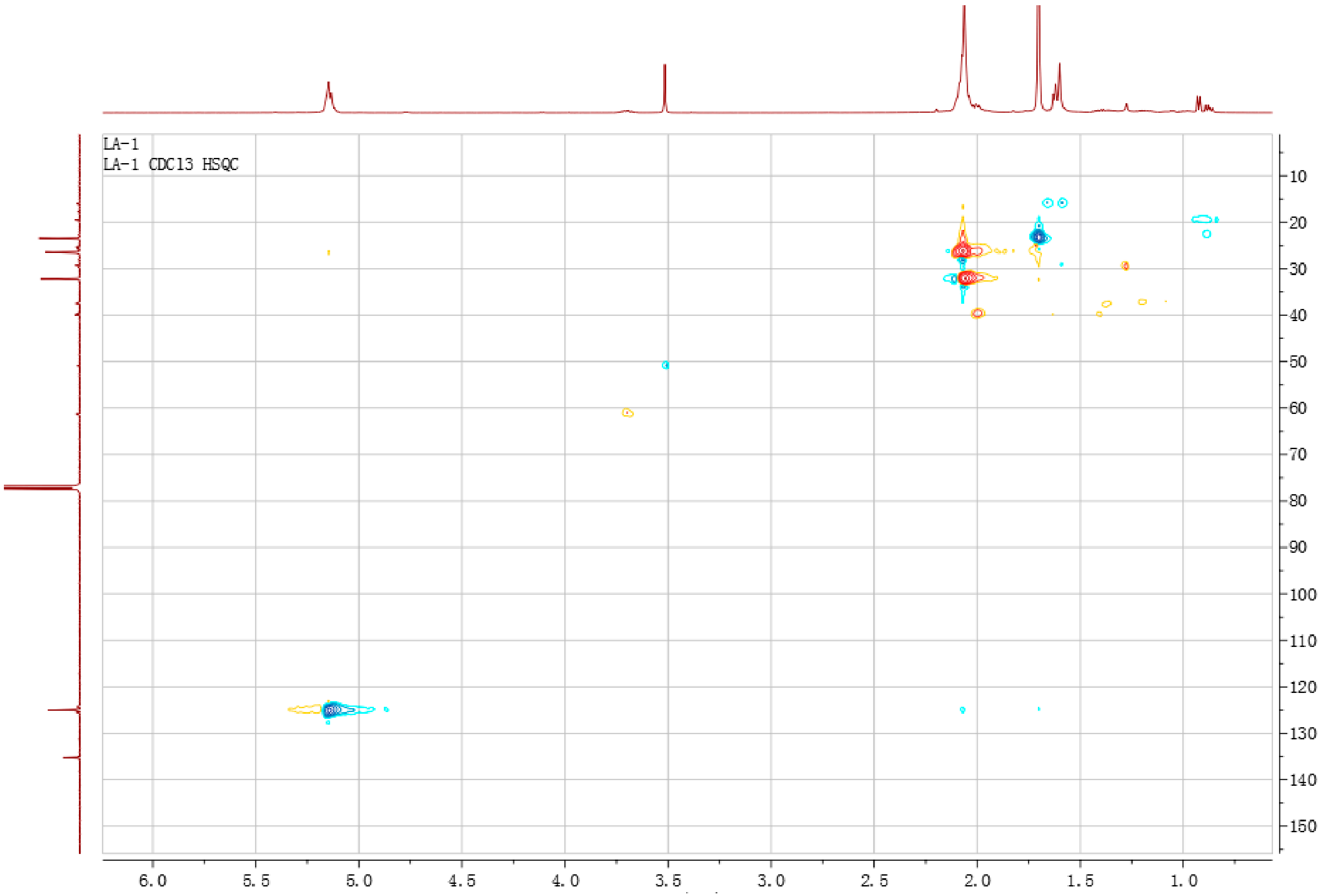

Figure S27. HSQC spectrum (500 MHz) of compound **3** in $CDCl_3$.

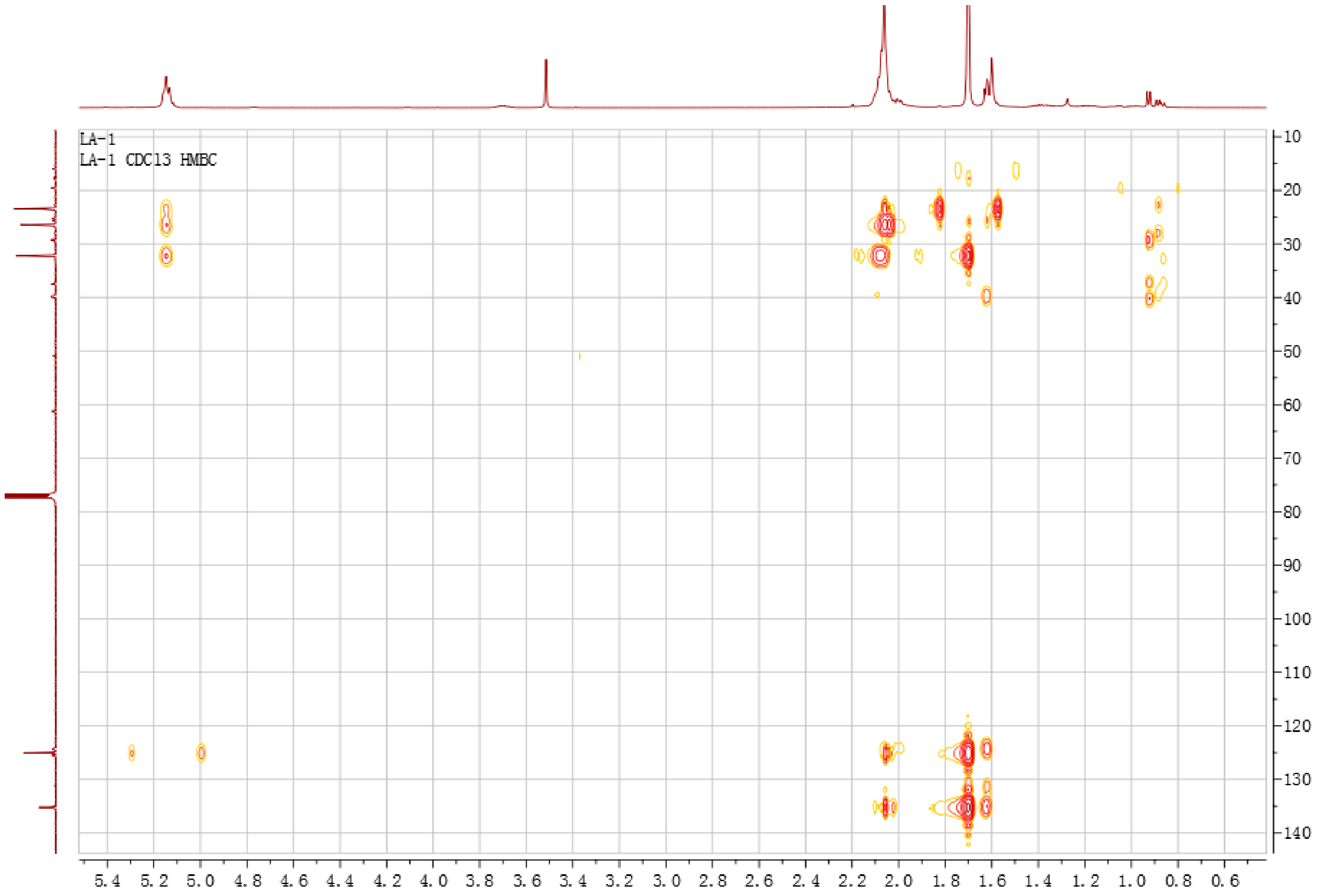

Figure S28. HMBC spectrum (500 MHz) of compound **3** in $CDCl_3$.

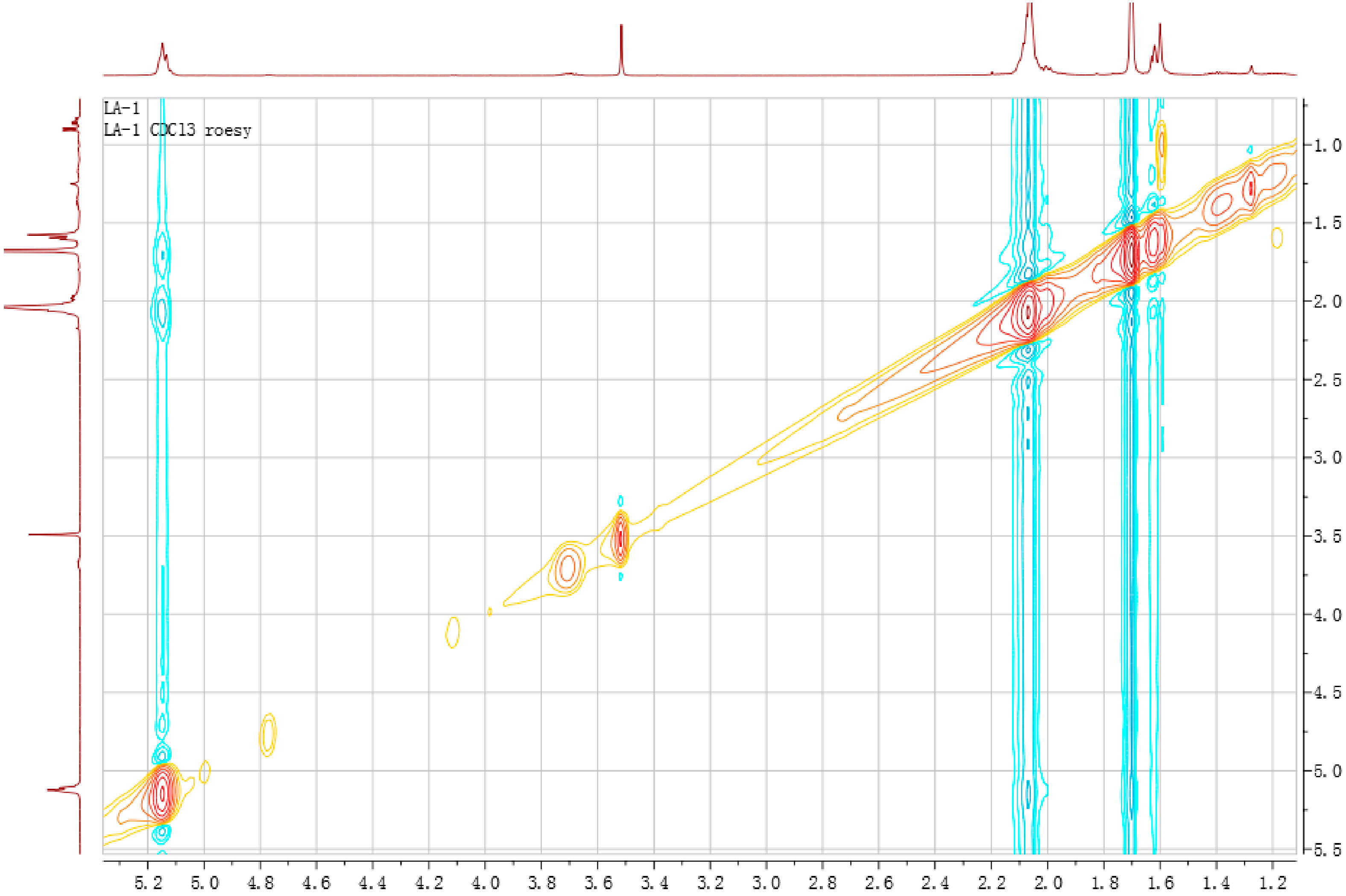

Figure S29. ROESY spectrum (500 MHz) of compound **3** in $CDCl_3$.

Reference

1. Frisch, M.J.; Trucks, G.W.; Schlegel, H.B.; Scuseria, G.E.; Robb, M.A.; Cheeseman, J.R.; Montgomery, J.A., Jr.; Vreven, T.; Kudin, K.N.; Burant, J.C.; *et al. Gaussian 03, Revision E.01*; Gaussian, Inc.: Wallingford, CT, USA, 2004.
2. Casida, M.E. *Recent Advances in Density Functional Methods, Part I*; Chong, D.P., Ed.; World Scientific: Singapore, 1995; pp. 155–192.
3. Gross, E.K.U.; Dobson, J.F.; Petersilka, M. Density functional theory of time-dependent phenomena. *Top. Curr. Chem.* **1996**, *181*, 81–172.
4. Gross, E.K.U.; Kohn, W. Time-dependent density functional theory. *Adv. Quantum Chem.* **1990**, 21, 255–291.
5. Runge, E.; Gross, E.K.U. Density-functional theory for time-dependent systems. *Phys. Rev. Lett.* **1984**, *52*, 997–1000.
6. Miertus, S.; Tomasi, J. Approximate evaluations of the electrostatic free energy and internal energy changes in solution processes. *Chem. Phys.* **1982**, *65*, 239–245.
7. Tomasi, J.; Persico, M. Molecular interactions in solution: An overview of methods based on continuous distributions of the solvent. *Chem. Rev.* **1994**, *94*, 2027–2094.
8. Cammi, R.; Tomasi, J. Remarks on the use of the apparent surface charges (ASC) methods in solvation problems: Iterative versus matrix-inversion procedures and the renormalization of the apparent charges. *J. Comp. Chem.* **1995**, *16*, 1449–1458.
